# Biological evaluation of semi-synthetic isoindolinone isomers produced by *Stachybotrys chartarum*

**DOI:** 10.3389/ffunb.2024.1494795

**Published:** 2024-11-22

**Authors:** Alica Fischle, Ulrich Schreiber, Viola Haupt, Felix Schimang, Lina Schürmann, Matthias Behrens, Florian Hübner, Melanie Esselen, Dmitrii V. Kalinin, Svetlana A. Kalinina

**Affiliations:** ^1^ Instititue of Food Chemistry, University of Münster, Münster, Germany; ^2^ Graduate School of Natural Products, University of Münster, Münster, Germany; ^3^ Institute of Pharmaceutical and Medicinal Chemistry, University of Münster, Münster, Germany

**Keywords:** semi-synthesis, regioisomerism, *Stachybotrys*, genotoxicity, biological activity, hepatic metabolism

## Abstract

The filamentous fungus *Stachybotrys chartarum* is rich in meroterpenoid secondary metabolites, some of which carry *o*-dialdehyde moieties, which are readily derivatized to isoindolinones by addition of primary amines. The structural diversity of phenylspirodrimanes, in particular, is linked to a wide range of biological activities, making them ideal candidates for semi-synthetic modification. In this study, acetoxystachybotrydial acetate was reacted with l-tryptophan and tryptamine, resulting in the detection of both regiospecific isomeric structures - a rare and significant finding that enabled the examination of four novel reaction products. Besides their successful purification, a detailed report on their isomer-specific behavior with regard to chromatographic retention, UV-spectral specificities, nuclear magnetic resonances, and mass spectrometric fragmentation is given. Furthermore, a comprehensive insight into each compounds’ unique effect within the tested biological assays is provided, which include cytotoxicity, genotoxicity, their biological activity against serine proteases of the blood coagulation cascade, and *in vitro* hepatic metabolism, always in comparison to the non-derivatized substance. Ultimately, each isomer can be distinguished already during the purification process, which extends to the biological assays where we present one less cytotoxic, faster metabolized, and more active regio-isomeric phenylspirodrimane-derivative.

## Graphical Abstract

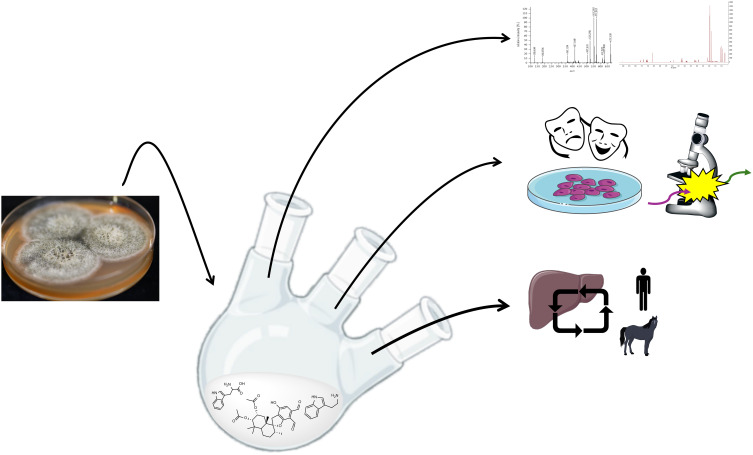

## 1 Introduction

**Figure 1:**
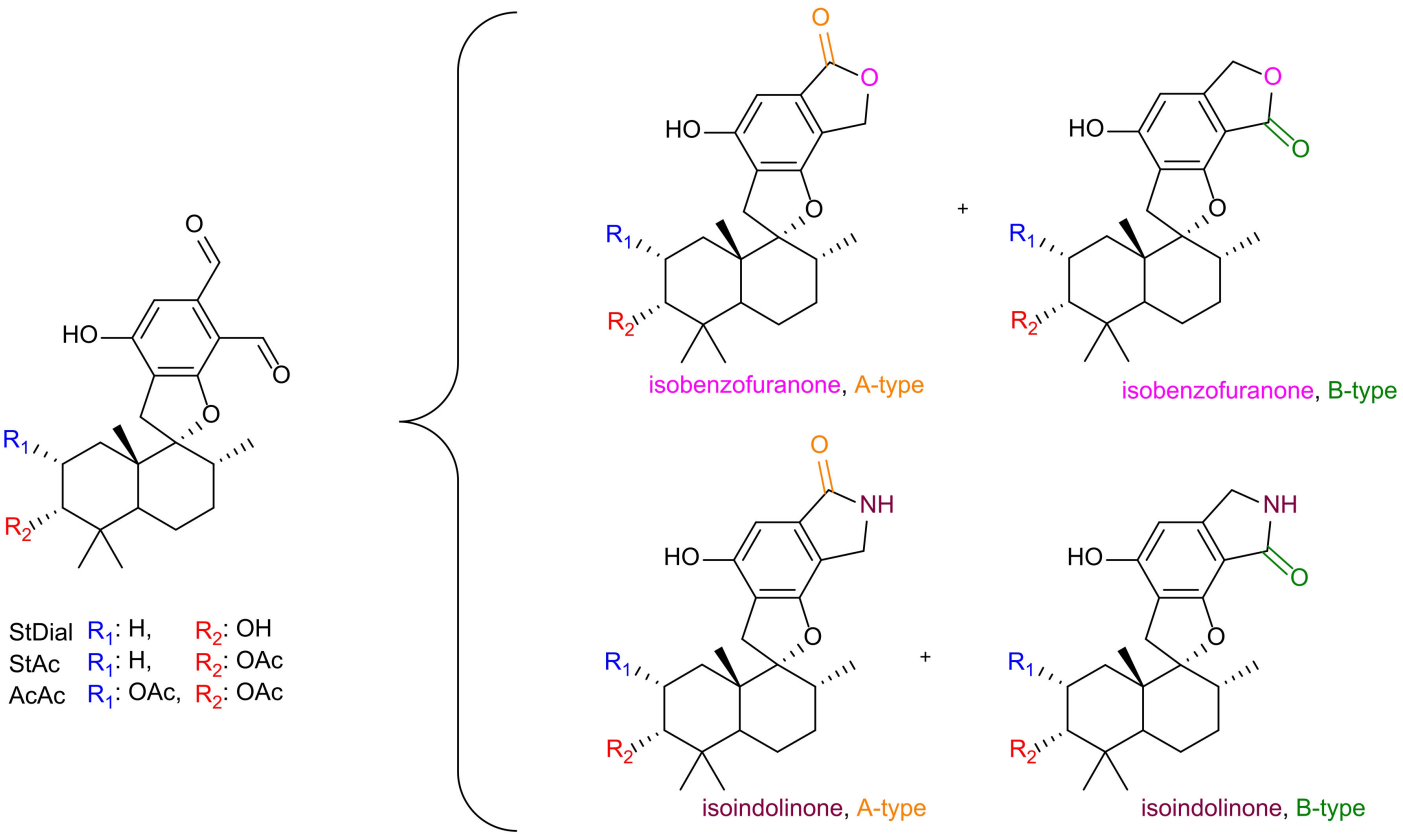
Overview of reaction products from *o*-dialdehydes. Identity of the PSDs is assigned by R_1_ (blue) and R_2_ (red). Incorporation of a lactone (pink oxygen) or lactam (purple nitrogen) with isomeric identity (A-type = orange, B-type = green).

Natural products (NPs) have long played a crucial role in novel drug discoveries (Newman and Cragg, 2020; Atanasov et al., 2021). Due to their complex chemical structures, NPs can exhibit multiple function within a single molecule, making them susceptible to modifications through metabolic processes and amenable to semi-synthetic alterations of reactive functional groups (Lourenço et al., 2012). Among them, phenylspirodrimanes (PSDs) produced by the filamentous fungi genus *Stachybotrys* provide a chemically versatile foundation for generating a wide array of biologically active compounds through semi-synthetic modifications. PSDs carry reactive *o*-dialdehyde groups, which naturally suggest a cyclization reaction *via* nucleophilic addition. Although this reaction has been observed in previous investigations, the exact mechanism remains elusive (Thiele and Schneider, 1909; Do Minh et al., 1977; Takahashi et al., 2005; Augner et al., 2011; D’Hollander and Westwood, 2018; Jagels et al., 2019). Depending on the electron-donating/-withdrawing properties of the substituent, two regiospecific isomeric forms may be generated: namely, type A (carbonyl group at position 8) or type B (carbonyl group at position 9) (D’Hollander and Westwood, 2018). This nucleophilic attack results in the formation of either a lactone (isobenzofuranone) or a lactam derivative (isoindolinone) as illustrated in **Figure 1**.

**Figure 2:**
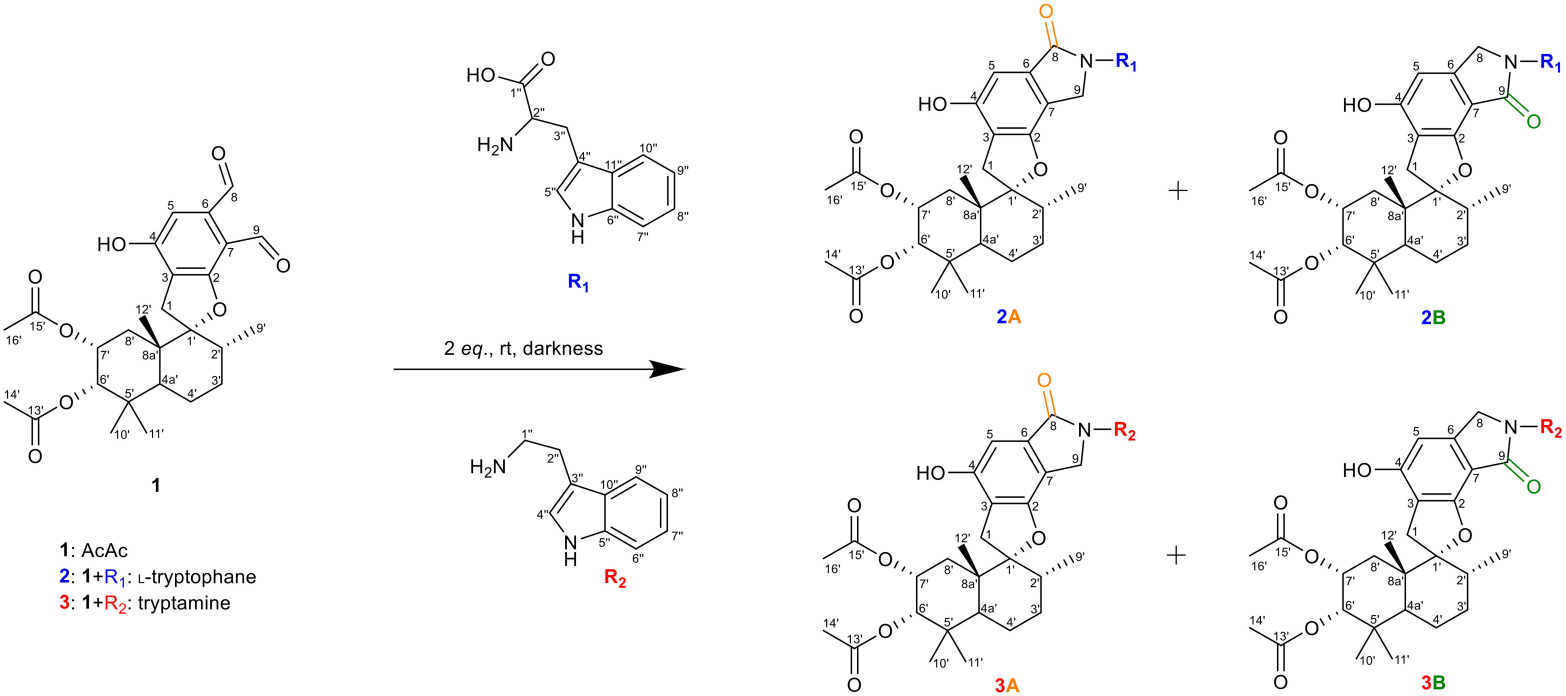
Overview of reaction. R_1_ is l-tryptophan (blue), R_2_ is tryptamine (red) with AcAc (black). Formation of lactam as A-type (orange) or B-type (green) depending on position of the carbonyl group. Carbon atoms numbering according to IUPAC.

Our initial investigations centered on three PSD compounds, stachybotrydial (StDial), stachybotrydial acetate (StAc), and acetoxystachybotrydial acetate (AcAc, ***1***) (**Figure 2**) to assess their potential impact on serine proteases of the blood coagulation cascade (Steinert et al., 2022). From a micro-scale screening of 35 primary amines, l-arginine and agmatine emerged as particularly promising candidates. Notably, the semi-synthetically derived compounds exhibited reduced cytotoxicity compared to their parent molecules and demonstrated *in vitro* activity against all tested serine proteases at a concentration of 200 µM. Among these, the agmatine-derivatives proved more potent than isoindolinones derived from arginine, which was attributed to the absence of the carboxyl group. Further evaluation of the most active compound, AcAc-agmatine, showed prolonged plasma coagulation in both the activated partial thromboplastin time and the prothrombin time, which confirmed the observed inhibition of Factor XIIa (intrinsic pathway) and Factor Xa (common pathway) (Steinert et al., 2022). Additionally, l-tryptophan, identified as another potentially active derivative in the micro-scale screening was reacted with AcAc (***1***) along with its primary amine, tryptamine, to further explore its bioactivity (**Figure 2**) to both acetoxystachybotrylactam acetate-tryptophan (***2***) and acetoxystachybotrylactam acetate-tryptamine (***3***).

In addition to processing the crude extracts to obtain highly pure reaction products presenting both isomeric structures (***2a***, ***2b***, ***3a***, ***3b***), we confirmed their structures using NMR and HRMS, followed by *in vitro* assessments of toxicity and biological activity. Since PSDs are highly associated with adverse effects especially on the respiratory system, with humans and horses being mainly affected (Drobotko, 1945; Harrach et al., 1983, 1987; Kaneto et al., 1994; Jarvis et al., 1995; Jarvis et al., 1998; Dearborn et al., 1999), additional investigations regarding the toxic potential of the starting materials, as well as experiments regarding the safety of the derivatized compounds were conducted. Here, the micronucleus assay was performed to assess genotoxicity (Alonso-Jauregui et al., 2021), while hepatic *in vitro* metabolism studies give first insights into possible detoxification reactions. Collectively, these investigations integrate a range of chemical, analytical, and biological approaches, allowing not only a comprehensive assessment of each compound’s properties but also a detailed comparison of the isomeric pairs.

## 2 Materials and methods

### 2.1 General devices, chemicals, and experimental procedures

Purity measurements of the isolated and synthesized compounds were performed on an HPLC-DAD-ELSD with LC PU-2089 system with UV detection (MD-2010) (Jasco, Groß-Umstadt, Germany) and evaporative light scattering detection (ELSD) (Shimadzu, Duisburg, Germany). A Nucleodur^®^ Phenyl-Hexyl column (250 x 4 mm, 5 µm) equipped with a 4 x 3 mm Phenyl-Hexyl guard column (Macherey-Nagel, Düren, Germany) was used for separation, employing a binary gradient consisting of MeCN and H_2_O (both with 0.1% FA). The gradient started with 5% B, was held for 1 min, increased to 80% B over 34 min, then was increased again to 95% B within 0.01 min, held for 2.5 min, and decreased again to starting conditions within 0.01 min. Re-equilibration occurred for 2.5 min. Total run time was 40 min, the flow was 1.4 mL/min, with oven temperature at 40 °C. The wavelength of the UV detector was set to 255 nm, DAD detection occurred from 195-650 nm. The ELSD was set to a temperature of 50 °C, gain was 10, and 350 kPa of compressed air was used. Purity results are given in the description of the compounds. For data acquisition and processing, ChromPass Chromatography (Version 1.8.6.1, Jasco, Groß-Umstadt, Germany) was utilized.

Full sets (^1^H, ^13^C, HMBC, HSQC, and COSY) of NMR spectra were recorded on an Agilent DD2 600 MHz spectrometer (Agilent Technologies, Waldbronn, Germany); chemical shifts (δ) are reported in ppm relative to the solvent peak or internal standard (TMS). Compounds were either dissolved in MeOH-*d_4_*, MeCN-*d_3_*, or pyridine-*d_5_*.

All chemicals were purchased in analytical grade from Sigma-Aldrich GmbH (Seelze, Germany), VWR (Darmstadt, Germany), Fisher Scientific (Schwerte, Germany), Grüssing GmbH (Filsum, Germany) or Carl Roth (Karlsruhe, Germany). Ultrapure water was generated using a PURELAB Flex 2 system (Veolia Water Technologies, Celle, Germany). Deuterated NMR-solvents were obtained from ARMAR Chemicals (Döttingen, Switzerland).

### 2.2 Fungal strains and cultivation conditions

In order to isolate higher quantities of phenylspirodrimanes, filamentous fungus *Stachybotrys chartarum* IBT 40288 strain (IBT culture collection of fungi, Technical University of Denmark, Denmark) was cultured. For liquid pre-cultures, an approximately 1 cm^2^ agar plug covered with the fungal strain was used to inoculate 100 mL of Darken medium (DVK; Darken et al., 1959) in an Erlenmeyer flask. Cultivation took place for 3 days at 30°C shaking at 180 rpm in the dark. Large-scale cultures were grown on potato-dextrose agar (PDA, 4 g/L potato infusion, 20 g/L dextrose, 15 g/L agar) for 21 days at 25 °C in the dark after inoculation with 500 µL from DVK pre-cultures. The isolation procedure as well as analytical characterization was performed according to the protocol established from our group (Steinert et al., 2022).

### 2.3 Semi-synthetic reaction of PSD with l-tryptophan

The semi-synthetic modification of acetoxystachybotrydial acetate (***1***) with l-tryptophan followed our group’s established procedure (Steinert et al., 2022). Briefly, reactant 1 (PSD, 1 *eq.* in MeCN) was mixed with reactant 2 (l-tryptophan, 2 *eq*. in H_2_O) in a 10 mL round-bottom flask. Reactant 2 was supplied dropwise to start the reaction and the mixture was stirred continuously for up to 4 h at room temperature in the dark. Before quenching the reaction by removal of the solvent, formation of reaction products was confirmed by measurement of a 1:250 diluted sample *via* HPLC-Orbitrap-HRMS.

### 2.4 Optimized reaction parameters and final semi-synthetic reaction of PSD with tryptamine

The reaction between PSD and tryptamine was optimized by investigating the use of aprotic solvents, the effect of acidification, reaction time, and slow-addition of the reactants. Three aprotic solvents were investigated, DCM, DMF, and DMSO. Acidification was examined by preparing four concentration levels (10, 33, 50, 100 µM) of glacial acetic acid in each aprotic solvent system. The reaction time was varied from 2 h to 6 h. Also, instead of dropwise addition of reactant 2, the primary amine was supplied by use of a syringe pump at a defined flow-rate. Finally, either reactant 1 (PSD) was supplied to reactant 2 (primary amine) or reactant 2 (primary amine) was given to reactant 1 (PSD). During each experimental set, a reaction at the previously presented conditions for l-tryptophan was performed as a control. The final procedure was performed in a three-necked 10 mL round-bottom flask. Here, reactant 1 (PSD, 1 *eq.* in DMSO with 100 µM of glacial acetic acid) was mixed with molecular sieve (3 Å) and a magnetic stirrer. The reaction was started by supplying reactant 2 (tryptamine, 2 *eq*. in DMSO) at a flow rate of 10 µL/min through a syringe directly inserted into the solvent of reactant 1. After 2 h, remnants of reactant 2 were added to the reaction manually. The reaction was performed for a total of 3 h under constant stirring at RT in the dark. Confirmation of reaction product formation was performed by measurement of a 1:200 diluted sample *via* HPLC-qToF-HRMS.

### 2.5 Analytical determination of reaction products

The exact *m/z* of the reaction products with l-tryptophan (***2a***, ***2b***) were confirmed using an LC-Orbitrap-HRMS system. Chromatographic separation was achieved with a binary gradient (MeCN and H_2_O, both with 0.1% FA) on a ReproSil Gold C18 column (150 x 2 mm, 3 μm) equipped with a guard column of the same material (5 x 2 mm, both Dr. Maisch, Ammerbuch, Deutschland). The gradient of MeCN + 0.1% FA (solvent A) and H_2_O + 0.1% FA (solvent B) started at 10/90 solvent A/solvent B (v/v) for 2 min and a flow rate of 0.3 mL/min. Within 20 min, A was raised linearly to 100% and held there for 5 min. Re-equilibration at 10% A was performed for additional 5 min. The oven temperature was 40 °C throughout. High-resolution mass spectrometric data for structural characterization were obtained on a LTQ Orbitrap XL™ mass spectrometer applying heated-electrospray ionization (HESI) (Thermo Fisher Scientific, Dreieich, Germany) operated in positive ionization mode coupled to a Shimadzu LC-system consisting of a DGU-20A5R degasser, Nexera XT LC-20AD XR pump, Nexera XR, SIL-20AC XR autosampler, CTO-10SD VP oven unit, connected through a CBM-20A communication bus module (all Shimadzu, Tokyo, Japan) to the HRMS instrument. The source voltage was set to 3.5 kV, while capillary and vaporizer temperatures were set to 350 °C. The gas flows were set to arbitrary units (aU). Here, sheath gas was 40 aU, auxiliary gas at 20 aU, and sweep gas at 5 aU. Full scans were recorded in profile mode with a resolution of 30,000 in a mass range of *m/z* 200-1000. For data acquisition and analysis, the Xcalibur 3.1 software (Thermo Fisher Scientific, Dreieich, Germany) was utilized.

For the semi-synthesis with tryptamine (***3a***, ***3b***), an HPLC-qToF-HRMS system was utilized. Chromatographic separation was realized using a Elute HPG 1300 HPLC system coupled to an Impact II qToF mass spectrometer (both Bruker Daltonics, Bremen, Germany). Here, chromatographic separation was achieved on a ReproSil Gold C18 (150 x 2 mm, 3 μm) column equipped with a guard column of the same material (5 x 2 mm, both Dr. Maisch, Ammerbuch, Germany). The gradient consisted of H_2_O + 0.1% FA (solvent A) and MeCN + 0.1% FA (solvent B). Initial conditions were at 10% B, which was held for 2 min. Within 20 min, B was increased to 100%, held for 3 min, decreased to starting conditions within 0.1 min and re-equilibrated for 1.9 min. Throughout the gradient, the flow was consistent at 0.3 mL/min, with the oven temperature at 40 °C. Ionization was achieved in ESI using an Apollo II ion source. For positive ionization, the capillary voltage was at 4.5 kV, negative ionization occurred at -4.5 kV. The source temperature was at 220 °C, with an endplate offset of 500 V and 2.0 bar nebulizer gas pressure and 10 L/min dry gas flow. Spectra were recorded in line and profile mode, the mass range scanned was *m/z* 100-1100 at a spectra rate of 2 x 6 Hz, Funnel 1 RF at 250 Vpp, Funnel 2 RF at 300 Vpp, hexapole RF at 300 Vpp, collision RF at 500 Vpp. In-source CID was off, ion energy was 5.0 eV, and collision energy was 10 eV. Transfer time was 120 µs with a 10 µs pre-pulse storage. Fragmentation occurred in DDA Auto-MS/MS mode. Automatic fragmentation was induced if a signal threshold (48 counts) was reached by applying 10.0 eV (collision radiofrequency 650.0 Vpp) to the compound in a collision-induced dissociation cell. Calibration was 10 mM sodium formate continuously infused for 2 min directly after each injection. For data acquisition and processing, the Compass HyStar 4.1, Compass oToF control 2.0, and Compass Data Analysis 4.4 (all Bruker Daltonics, Bremen, Germany) and OriginPro2024 (Version 10.1.0.170, OriginLab Corporation, Northampton, MA, USA) were utilized.

### 2.6 Purification, purity determination, and structure confirmation

Further purification of the tryptophan-derivatives (***2a***, ***2b***) was performed on a semi-preparative HPLC-UV system which included a 4-Channel Vacuum in-line Degasser (EGG 102.107, TechLab GmbH, Braunschweig, Germany), two PU-2087 pumps (Jasco, Groß-Umstadt, Germany) connected to a Rheodyne 7125 manual injector (optiLab Chromatographietechnik GmbH, Berlin, Germany) and a UV-2075 detector (Jasco, Groß-Umstadt, Germany), all components were connected by a LC-NetII/ADC connector (Jasco, Groß-Umstadt, Germany). For data acquisition and processing, ChromPass Chromatography (Version 1.8.6.1, Jasco, Groß-Umstadt, Germany) was utilized. The reaction product was dissolved in MeCN/H_2_O (8/2, v/v) and separated chromatographically on a ZORBAX Eclipse XDB-C18 column (250 x 9.4 mm, 5 µm, Agilent, Waldbronn, Germany) equipped with a C18 guard cartridge (4 x 3 mm, Phenomenex, Aschaffenburg, Germany) with a binary gradient consisting of H_2_O + 0.1% FA (solvent A) and MeCN + 0.1% FA (solvent B) at a flow rate of 5.0 mL/min. The gradient started at 5% B, increased to 55% B within 4 min, was held there for 3 min, increased to 63% B within 9 min, driven to 95% B within 0.1 min, and held there for 1.9 min. Then, the gradient was decreased immediately to 5% B and the column was re-equilibrated at starting conditions for 4 min. The monitored wavelength was at 255 nm, ***2a*** and ***2b*** eluted at 12.7 min and 8.8 min, respectively.

Directly after the reaction of PSD with tryptamine (***3a***, ***3b***), a wash-out of DMSO and glacial acetic acid was performed. Here, the reaction mixture was diluted to approx. 1% DMSO in H_2_O and loaded onto a 5 g/20 mL Strata^®^ C18-E cartridge under reduced pressure. After loading approx. 1/3rd of the diluted mixture, a washing step was performed with H_2_O before continuing loading of the sample. This intermediate washing step was repeated again after loading 2/3rd of the sample and after completion of sample loading. Elution of the reaction products was started with MeCN/H_2_O (4/6, v/v) by increasing the organic content in steps of 10% until 100% MeCN. Each elution step had a volume of 50 mL. All fractions were collected, combined, brought to dryness, reconstituted in MeCN, and submitted for semi-preparative HPLC-DAD-UV. The system contained two LC-20AT pumps, a CTO-20AS VP column oven, an SPD-M20A prominence diode array detector connected by a CBM-20A prominence communication bus module (all Shimdazu, Tokyo, Japan). Injection occurred with a Varian ProStar manual injector (Varian Analytical Instruments, Walnut Creek, CA, USA). For data processing, LCsolution (version 1.25, Shimadzu, Tokyo, Japan) was utilized. Chromatographic separation was achieved on a Nucleodur Phenyl-Hexyl column (250 x 9.4 mm, 5 µm, Macherey-Nagel, Düren, Germany) equipped with a Phenyl-Hexyl guard column (4 x 3 mm, Phenomenex, Aschaffenburg, Germany) with a binary gradient consisting of H_2_O (solvent A) and MeCN (solvent B) at a flow rate of 5.0 mL/min. The gradient started at 72% B and increased to 75% B in 8 min, was driven to 100% B within 0.1 min, held for 1 min, decreased again to 72% B within 0.1 min, and re-equilibrated for 4 min. For UV-detection, 255 nm and 290 nm were monitored, while DAD spectra were recorded from 195-800 nm at 1.56 Hz. Compound ***3a*** and ***3b*** eluted at 6.4 min and 5.1 min, respectively.

The fractions containing the reaction products were combined and evaporated *in vacuo*, underwent purity determination, and further structural characterization according to the descriptions above.

For fragmentation experiments, the previously described HPLC-qToF-system was used. Here, the compounds were injected as a standard solution at either 10 µM or 1 µg/mL in MeCN/H_2_O (8/2, v/v). Chromatographic separation and HRMS detection occurred as previously described.

*Acetoxystachybotrylactam acetate-tryptophan (* ***2a*** *).* Colorless powder (4.6 mg, 14.2%). UV *λ_max_* (MeCN/H_2_O + 0.1% FA): 223, 267 nm. ^1^H NMR (600 MHz, CD_3_OD) *δ* 10.12 (s, 1H, NH), 9.21 (s, 1H, OH^’’^), 8.08 (s, 1H, OH^’’^), 7.61 (d, *J* = 7.9 Hz, 1H, H-10^’’^), 7.27 (d, *J* = 8.0 Hz, 1H, H-7^’’^), 7.05 (dd, *J* = 7.1, 0.9 Hz, 1H, H-8^’’^), 7.03 (s, 1H, H-5^’’^), 6.98 (td, *J* = 7.4, 1.0 Hz, 1H, H-9^’’^), 6.64 (s, 1H, H-5), 5.26 (s, 1H, H-2^’’^), 5.20 (ddd, *J* = 12.8, 4.5, 2.5 Hz, 1H, H-7^’^), 4.92 (d, *J* = 2.4 Hz, 1H, H-6^’^), 4.54 (d, *J* = 16.5 Hz, 1H, H-9), 4.34 (d, *J* = 16.4 Hz, 1H, H-9), 3.68-3.54 (m, 1H, H-3^’’^), 3.50-3.40 (m, 1H, H-3^’’^), 3.18 (d, *J* = 17.1 Hz, 1H, H-1), 2.87 (d, *J* = 17.1 Hz, 1H, H-1), 2.11 (dd, *J* = 12.2, 2.8 Hz, 1H, H-4a^’^), 2.07 (s, 3H, H-14^’^), 2.03 (s, 1H, H-4-OH), 1.87-1.84 (m, 3H, H-2^’^, H-16^’^), 1.80 (t, *J* = 12.4 Hz, 1H, H-8^’^), 1.65-1.55 (m, 2H, H-3^’^, H-4^’^), 1.53-1.42 (m, 2H, H-3^’^, H-4^’^), 1.34 (dd, *J* = 12.0, 4.5 Hz, 1H, H-8^’^), 1.13 (s, 3H, H-12^’^), 1.03 (s, 3H, H-11^’^), 0.92 (s, 3H, H-10^’^), 0.69 (d, *J* = 6.6 Hz, 3H, H-9^’^). ^13^C NMR (150 MHz, DMSO-*d_6_*) *δ* 205.4 (C, C-1^’’^), 172.8 (C, C-13^’^), 172.4 (C, C-15^’^), 171.5 (C, C-8), 157.1 (C, C-2), 155.3 (C, C-4), 138.0 (C, C-6^’’^), 135.2 (C, C-6), 128.7 (C, C-11^’’^), 123.6 (CH, C-5^’’^), 122.4 (CH, C-8^’’^), 119.8 (CH, C-9^’’^), 119.2 (CH, C-10^’’^), 118.3 (C, C-3), 114.6 (C, C-7), 112.3 (CH, C-7^’’^), 108.8 (C, C-4^’’^), 102.5 (CH, C-5), 99.1 (C, C-1^’^), 78.3 (CH, C-6^’^), 69.7 (CH, C-7^’^), 49.6 (CH, C-2^’’^), 46.1 (CH_2_, C-9), 44.9 (C, C-8a^’^), 41.8 (CH, C-4a^’^), 39.1 (C, C-5^’^), 37.7 (CH, C-2^’^), 32.9 (CH_2_, C-1), 32.1 (CH_2_, C-3^’^), 31.5 (CH_2_, C-8^’^), 28.4 (CH_3_, C-10^’^), 27.3 (CH_2_, C-3^’’^), 22.0 (CH_3_, C-11^’^), 21.5 (CH_2_, C-4’), 21.2 (CH_3_, C-14^’^), 21.0 (CH_3_, C-16^’^), 17.2 (CH_3_, C-12^’^), 15.8 (CH_3_, C-9^’^).

HRMS (10 µM): *m/z* 673.3124 (calc for [C_38_H_44_N_2_O_9_]^+^ 673.3120, *Δm*: -0.6 ppm), purity ≥ 99%.

*Acetoxystachybotrylactam acetate-tryptophan (* ***2b*** *).* Colorless powder (8.9 mg, 27.5%). UV *λ_max_* (MeCN/H_2_O + 0.1% FA): 203, 227, 259, 291 nm. ^1^H NMR (600 MHz, CD_3_OD) *δ* 8.42 (s, 1H, OH^’’^), 7.61 (d, *J* = 8.1 Hz, 1H, H-10^’’^), 7.30 (d, *J* = 8.1 Hz, 1H, H-7^’’^), 7.06 (ddd, *J* = 8.0, 7.0, 1.1 Hz, 1H, H-8^’’^), 7.04 (s, 1H, H-5^’’^), 6.97 (ddd, *J* = 7.9, 7.0, 1.0 Hz, 1H, H-9^’’^), 6.30 (s, 1H, H-5), 5.17 (ddd, *J* = 12.7, 4.6, 2.4 Hz, 1H, H-7^’^), 4.84 (s, 1H, H-2^’’^), 4.77 (s, 1H, H-6^’^), 4.47 (d, *J* = 16.8 Hz, 1H, H-8), 4.34 (d, *J* = 16.8 Hz, 1H, H-8), 3.61-3.54 (m, 1H, H-3^’’^), 3.33-3.32 (m, 1H, H-3^’’^), 3.07 (d, *J* = 16.4 Hz, 1H, H-1), 2.81 (d, *J* = 16.3 Hz, 1H, H-1), 2.33 (dd, *J* = 12.7, 2.8 Hz, 1H, H-4a^’^), 1.88-1.86 (m, 1H, H-2^’^), 1.85 (s, 3H, H-16^’^), 1.80 (t, *J* = 12.3 Hz, 2H, H-8^’^), 1.75-1.66 (m, 2H, H-3^’^), 1.70 (s, 3H, H-14^’^), 1.58 (t, *J* = 12.0 Hz, 4H, H-3^’^, H-4^’^), 1.47 (qd, *J* = 13.3, 4.2 Hz, 2H, H-4^’^), 1.29 (q, *J* = 4.8 Hz, 2H, H-8^’^), 1.12 (s, 3H, H-12^’^), 1.01 (s, 3H, H-11^’^), 0.90 (s, 3H, H-10^’^), 0.78 (d, *J* = 6.5 Hz, 3H, H-9^’^).

^13^C NMR (150 MHz, CD_3_OD) *δ* 173.2 (C, C-13^’^), 172.4 (C, C-15^’^), 169.7 (C, C-9), 159.8 (C, C-2), 158.6 (C, C-4), 146.7 (C, C-7), 138.1 (C, C-6^’’^), 128.7 (C, C-11^’’^), 123.7 (CH, C-5^’’^), 122.5 (CH, C-8^’’^), 119.8 (CH, C-9^’’^), 119.2 (CH, C-10^’’^), 113.9 (C, C-3), 112.3 (CH, C-7^’’^), 107.33 (C, C-6), 102.61 (CH, C-5), 99.79 (C, C-1^’^), 78.45 (CH, C-6^’^), 70.02 (CH, C-7^’^), 49.62 (CH_2_, C-8), 47.2 (C, C-2^’’^), 44.9 (C, C-8a^’^), 41.1 (CH, C-4a^’^), 39.2 (C, C-5^’^), 37.6 (CH, C-6^’^), 31.8 (CH_2_, C-1), 31.8 (CH_2_, C-3^’^) 31.5 (CH_2_, C-8^’^), 28.2 (CH_3_, C-11^’^), 27.1 (CH_2_, C-3^’’^), 21.9 (CH_3_, C-10^’^), 21.6 (CH_2_, C-4^’^), 21.0 (CH_3_, C-16^’^), 20.7 (CH_3_, C-14^’^), 17.3 (CH_3_, C-12^’^), 15.8 (CH_3_, C-9^’^).

HRMS (10 µM): *m/z* 673.3126 (calc for [C_38_H_44_N_2_O_9_]^+^ 673.3120, *Δm*: -0.9 ppm), purity ≥ 99%.

*Acetoxystachybotrylactam acetate-tryptamine (* ***3a*** *).* Colorless powder (4.1 mg, 10.5%). UV *λ_max_* (MeCN/H_2_O): 213, 266 nm. ^1^H NMR (600 MHz, CD_3_CN) *δ* 9.08 (s, 1H, NH), 7.59 (dd, *J* = 7.9, 1.0 Hz, 1H, H-9^’’^), 7.37 (dt, *J* = 8.2, 0.9 Hz, 1H, H-6^’’^), 7.12 (ddd, *J* = 8.2, 7.0, 1.2 Hz, 1H, H-7^’’^), 7.10 (d, *J* = 2.5 Hz, 1H, H-4^’’^), 7.03 (ddd, *J* = 8.1, 7.0, 1.0 Hz, 1H, H-8^’’^), 6.62 (s, 1H, H-5), 5.15 (ddd, *J* = 12.8, 4.6, 2.6 Hz, 1H, H-7^’^), 4.87 (dd, *J* = 2.6, 1.0 Hz, 1H, H-6^’^), 4.28 (d, *J* = 16.5 Hz, 1H, H-9), 4.20 (d, *J* = 16.4 Hz, 1H, H-9), 3.88 (ddd, *J* = 14.3, 7.7, 6.7 Hz, 1H, H-1^’’^), 3.81 (ddd, *J* = 13.7, 7.8, 7.0 Hz, 1H, H-1^’’^), 3.18 (d, *J* = 17.0 Hz, 1H, H-1), 3.13-3.03 (m, 2H, H-2^’’^), 2.89 (d, *J* = 17.1 Hz, 1H, H-1), 2.06-2.00 (m, 1H, H-4a^’^), 1.93 (s, 3H, H-14^’^), 1.94-1.83 (m, 1H, H-2^’^), 1.80 (s, 3H, H-16^’^), 1.74 (t, *J* = 12.1 Hz, 1H, H-8^’^), 1.66-1.59 (m, 1H, H-3^’^), 1.59-1.53 (m, 1H, H-4^’^), 1.52-1.39 (m, 2H, H-3^’^, H-4^’^), 1.36 (dd, *J* = 11.9, 3.9 Hz, 1H, H-8^’^), 1.10 (s, 3H, H-12^’^), 1.01 (s, 3H, H-11^’^), 0.89 (s, 3H, H-10^’^), 0.72 (d, *J* = 6.5 Hz, 3H, H-9^’^).^13^C NMR (150 MHz, CD_3_CN) *δ* 171.4 (C, C-13^’^), 171.0 (C, C-15^’^), 168.7 (C, C-8), 156.9 (C, C-2), 154.4 (C, C-4), 137.5 (C, C-5^’’^), 136.1 (C, C-6), 128.4 (C, C-10^’’^), 123.4 (CH, C-4^’’^), 122.5 (CH, C-7^’’^), 119.8 (CH, C-8^’’^), 119.3 (CH, C-9^’’^), 117.4 (C, C-3), 114.1 (C, C-7), 113.3 (CH, C-6^’’^), 112.3 (C, C-3^’’^), 102.0 (CH, C-5), 98.9 (C, C-1^’^), 77.4 (CH, C-6^’^), 68.9 (CH, C-7^’^), 47.7 (CH_2_, C-9), 44.4 (CH_3_, C-8a^’^), 43.6 (CH_2_, C-1^’’^), 41.5 (CH, C-4a^’^), 38.8 (C, C-5^’^), 37.2 (CH, C-2^’^), 32.6 (CH_2_, C-1), 31.7 (CH_2_, C-3^’^), 31.1 (CH_2_, C-8^’^), 28.2 (CH_3_, C-10^’^), 24.9 (CH_2_, C-2^’’^), 21.8 (CH_3_, C-11^’^), 21.1 (CH_3_, C-16^’^), 21.1 (CH_2_, C-4^’^/CH_3_, C-14^’^), 17.1 (CH_3_, C-12^’^), 15.7 (CH_3_, C-9^’^).

HRMS (1 µg/mL): *m/z* 629.3226 (calc for [C_37_H_44_N_2_O_7_]^+^ 629.3222, *Δm*: -0.6 ppm), purity ≥ 97%.

*Acetoxystachybotrylactam acetate-tryptamine (* ***3b*** *).* Colorless powder (4.7 mg, 12.2%). UV *λ_max_* (MeCN/H_2_O): 219, 257, 290 nm. ^1^H NMR (600 MHz, C_5_D_5_N) *δ* 12.39 (s, 1H, OH), 11.81 (s, 1H, NH), 8.00 (dd, *J* = 6.9, 1.7 Hz, 1H, H-9^’’^), 7.61-7.55 (m, 1H, H-6^’’^), 7.32 (d, *J* = 2.0 Hz, 1H, H-4^’’^), 7.30 (dd, *J* = 7.7, 1.6 Hz, 1H, H-7^’’^), 7.28 (dd, *J* = 7.1, 1.4 Hz, 1H, H-8^’’^), 6.64 (s, 1H, H-5), 5.48 (ddd, *J* = 12.8, 4.6, 2.5 Hz, 1H, H-7^’^), 5.29 (d, *J* = 2.3 Hz, 1H, H-6^’^), 4.19 (d, *J* = 16.7 Hz, 1H, H-8), 4.06 (d, *J* = 16.6 Hz, 1H, H-8), 4.01 (dd, *J* = 8.5, 6.7 Hz, 2H, H-1^’’^), 3.50 (d, *J* = 16.2 Hz, 1H, H-1), 3.21 (dd, *J* = 8.5, 6.7 Hz, 2H, H-2^’’^), 3.13 (d, *J* = 16.3 Hz, 1H, H-1), 2.63 (dd, *J* = 12.8, 2.7 Hz, 1H, H-4a^’^), 2.45 (t, *J* = 12.4 Hz, 1H, H-8^’^), 2.38 (s, 3H, H-14^’^), 1.83 (s, 3H, H-16^’^), 1.82 (d, *J* = 4.6 Hz, 1H, H-3^’^), 1.76 (dtd, *J* = 12.5, 6.2, 4.1 Hz, 1H, H-2^’^), 1.63 (dd, *J* = 11.8, 4.5 Hz, 1H, H-8^’^), 1.58-1.47 (m, 2H, H-3^’^, H-4^’^), 1.33 (dd, *J* = 13.1, 4.1 Hz, 1H, H-4^’^), 1.07 (s, 3H, H-12^’^), 0.95 (s, 3H, H-11^’^), 0.91 (d, *J* = 6.3 Hz, 3H, H-10^’^), 0.89 (s, 3H, H-9^’^). ^13^C NMR (150 MHz, C_5_D_5_N) *δ* 171.4 (C, C-13^’^), 170.8 (C, C-15^’^), 167.4 (C, C-9), 159.5 (C, C-2), 158.7 (C, C-4), 145.9 (C, C-7), 138.2 (C, C-5^’’^), 128.9 (C, C-10^’’^), 123.6 (CH, C-4^’’^), 122.4 (CH, C-7^’’^), 119.7 (CH, C-8^’’^, C-9^’’^), 114.1 (C, C-3), 113.4 (CH, C-6^’’^), 112.5 (C, C-3^’’^), 108.2 (C, C-6), 102.9 (CH, C-5), 98.8 (C, C-1^’^), 77.5 (CH, C-6^’^), 69.2 (CH, C-7^’^), 50.7 (C, C-8), 44.5 (C, C-8a^’^), 43.6 (CH_2_, C-1^’’^), 40.9 (CH, C-4a^’^), 38.8 (C, C-5^’^), 37.3 (CH, C-2^’^), 32.5 (CH_2_, C-1), 31.5 (CH_2_, C-8^’^), 31.3 (CH_2_, C-3^’^), 28.3 (CH_3_, C-10^’^), 25.7 (CH_2_, C-2^’’^), 21.9 (CH_3_, C-11^’^), 21.4 (CH_3_, C-16^’^), 21.4 (CH_3_, C-14^’^), 21.1 (CH_2_, C-4^’^), 17.2 (CH_3_, C-12^’^), 16.2 (CH_3_, C-9^’^).

HRMS (1 µg/mL): *m/z* 629.3219 (calc for [C_37_H_44_N_2_O_7_]^+^ 629.3222, *Δm*: 0.5 ppm), purity ≥ 99%.

### 2.7 Cytotoxicity

The cytotoxic effects of the semi-synthesized products were evaluated with the resazurin assay, performed analogous to previous studies (Nociari et al., 1998; O’Brien et al., 2000). Human liver carcinoma cells (HepG2, HB-8065) and lung adenocarcinoma cells (A549, CCL-185) cells were cultivated in accordance with the descriptions of Kalinina et al. (Kalinina et al., 2018). The cells were seeded with 25,000 cells/well (HepG2) and 10,000 cells/well (A549) in 96-well plates and incubated for 24 h. After replacing the medium with a serum-free medium the cells were incubated for additional 24 h. The compounds of interest were applied in a concentration range of 0.1-100 µM and incubated for 24 h at 37 °C and 5% CO_2_. Afterwards, 10 µL of the standard resazurin solution was added to the cells and incubated for 2 h as previously described. The reduction of resazurin to resorufin was analyzed by screening the fluorescence at λ = 544 nm excitation and λ = 590 nm emission with a microplate reader (Infinite M200PRO, Tecan, Männendorf, Switzerland). Cytotoxicity assays were performed with three triplicates from three independent passages (*N*≥9) for ***2a***, ***2b***, while ***3a***, ***3b*** had six replicates from three independent passages (*N*≥18). Previously isolated T-2 toxin served as positive control in a concentration of 10 µM (Beyer et al., 2009). After subtraction of cell-free blank values, cellular viability was calculated as test-over-control (T/C) for each of the three passages. The data are shown as the mean ± standard deviation (SD). For detection of outliers, Grubb’s test was applied to all passages at 0.05 (two-sided) significance. Concentration dependent effect was evaluated by analysis of variance (one-way ANOVA) and the Tukey’s *post hoc* test (^*^p ≤ 0.05, ^**^p ≤ 0.01, ^***^p ≤ 0.001). The indicated significance represents the significance level relative to the solvent-treated control group (1% or 0.5% DMSO) calculated with the OriginPro2024 (Version 10.1.0.170, OriginLab Corporation, Northampton, MA, USA). The cell lines presented in this study were obtained from Merck (Darmstadt, Germany).

### 2.8 Micronucleus assay

The genotoxic effects of the AcAc (***1***) and its semi-synthetic products (***2a***, ***2b***; ***3a***, ***3b***) were evaluated with the micronucleus assay, performed based on OECD Test guideline 487. HepG2 were cultivated in accordance with the descriptions of Kalinina et al. (Kalinina et al., 2018). The cells were seeded with 300,000 cells/slide on Polysine^®^ slides (Epredia™ Polysine™ Microscopic Adhesion Slides, FisherScientific) in quadriPERM^®^ culture dishes (Sarstedt, Nümbrecht, Germany) and cultivated for 24 h. Then, they were incubated with the test compounds (0.5-25 µM) in serum free DMEM for 5 h. As positive control 0.6 µM mitomycin C (MMC) was used. All tested compounds were dissolved in DMSO. Cells were washed with sterile phosphate-buffered saline (PBS) and incubated with 4 µg/mL cytokinesis inhibitor cytochalasin B (Santa Cruz Biotechnology, USA) in DMEM for 19 h. Slides were washed with PBS and cold MeOH. Afterwards, cells were fixated with MeOH at -20 °C for 1 h. The slides were randomized and stained with 1 µg/mL 4′,6-diamidino-2-phenylindole and propidium iodide (Santa Cruz Biotechnology, USA) in antifade solution (0.1% w/v *p*-phenylendiamin dihydrochloride in 90% glycerol, 10% PBS, pH 8). Subsequently, cells were evaluated using a fluorescence microscope (Zeiss Axio Imager 2, Oberkochen, Germany) with filters of 365/445 nm and 546/575 nm. The micronucleus rate was evaluated by counting 1,000 binucleate cells and binucleate cells with micronuclei on each slide and calculating the proportion of binucleate cells containing one or more micronuclei. Genotoxicity assays were performed with cells from three independent passages (*N* = 3).

### 2.9 Serine protease inhibition assay

The inhibitory activity of further semi-synthesized compounds toward the coagulation factor XIIa, thrombin, FXIa, FXa, and trypsin was measured by quantifying the hydrolysis rate of the fluorogenic substrates as reported previously (Korff et al., 2020; Platte et al., 2021; Imberg et al., 2022). Briefly, the activity was tested in buffer (10 mM Tris-Cl, 150 mM NaCl, 10 mM MgCl_2_·6 H_2_O, 1 mM CaCl_2_·2 H_2_O, 0.1% w/v BSA, 0.01% v/v Triton-X100, pH 7.4) utilizing clear flat-bottom, black polystyrene 96-well plates. The enzymes (human β-FXIIa, HFXIIAB, > 95% purity; Molecular Innovations, 2.5 nM – final concentration; human α-thrombin (active) protein, ABIN2127880, > 95% purity; antibodies-online, 0.25 nM – final concentration; human Factor Xa, HFXA, > 95% purity; Molecular Innovations, 2.5 nM – final concentration; human factor XIa, HFXIA, >95% purity; Molecular Innovations, 0.5 nM – final concentration porcine trypsin; Merck, 3.5 nM – final concentration) and the fluorogenic substrates for FXIIa: Boc-Gln-Gly-Arg-AMC (Pepta Nova, 25 µM – final concentration, K_m_ = 167 µM); for thrombin: Boc Val Pro Arg AMC (Pepta Nova, 25 µM – final concentration, K_m_ = 18 µM); for FXa: Boc-Ile-Glu-Gly-Arg-AMC (Pepta Nova, 25 µM – final concentration); for FXIa: Boc-Glu-(OBzl)-Ala-Arg-AMC (Pepta Nova, 25 µM – final concentration); for trypsin: Z-Gly-Gly-Arg-AMC (Sigma-Aldrich, 25 µM – final concentration) were used. The fluorogenic substrate solution was added into the wells followed by the addition of test-compounds solution, and the reaction was triggered by addition of the enzyme solution (final testing volume – 150 µL). In case of blank (substrate + buffer) and control (substrate + enzyme) wells, DMSO was added instead of the test-compounds’ solution. Fluorescence intensity was measured with Microplate Reader Mithras LB 940 (Berthold Technologies, excitation at 355 nm, emission at 460 nm) for a period of 1 h with a read every minute. The reactions were performed at 25 °C. To derive % of enzyme inhibition, endpoint RFU (single fluorescence reading after 1 h) was used. As a positive controls for thrombin, FXa, FXIIa, FXIa, and trypsin were used dabigatran (500 nM), rivaroxaban (500 nM), compound “39b” (500 nM) (Platte et al., 2021), Inhibitor 5t (8 µM) (Imberg et al., 2022), Inhibitor E (2 µM) (Erbacher et al., 2024), respectively. Semi-synthesized compounds were screened at 200 µM.

### 2.10 Hepatic metabolism studies

Liver microsomes and cytosol were obtained for two organisms, horse and human. Fresh horse liver was purchased locally cut to pieces of ca. 50 g each and frozen in liquid nitrogen. Until further usage, the liver was stored at -80 °C. The preparation of microsomes and cytosol was carried out according to Balk et al. (Balk et al., 1984), and the protein content was determined by Bradford assay with bovine serum albumin as a reference (Bradford, 1976). Human liver microsomes and cytosol (150 Donors Ultrapool™) were provided by Corning, Inc. (Corning, New York, NY, USA). Besides assay-specific positive controls, all assays included stability controls, moreover, matrix influences were considered. For oxidation, additional non-oxidation controls were included. Each experiment was performed in duplicate. All stated concentrations are final concentration values. The test compounds (acetoxystachybotrylactam acetate-tryptophan ***2a, 2b*** and acetoxystachybotrylactam acetate-tryptamine ***3a, 3b***) were extended by the most biologically active derivative previously characterized by our group (acetoxystachybotrylactam acetate-agmatine ***4***) (Steinert et al., 2022).

The phase I metabolism was investigated utilizing a NADPH- regenerating system. The reaction mixture was prepared from ultrapure water, KH_2_PO_4_ (100 mM), NADP^+^ (664 µM), glucose-6-phosphate (G6P, 10 mM), G6P-dehydrogenase buffer (contains 10 mM Tris-base, 1 mM EDTA, 20 % glycerin, pH 7.4; final concentration 2 U/mL), the substance of interest (10 µM) and horse/human microsome (5 mg protein/mL). First, ultrapure H_2_O and KH_2_PO_4_ were added to the reaction vessel. Then, the substance to be investigated was added, followed by the regenerating system (NADP^+^, G6P, G6P-dehydrogenase), and finalized by addition of the microsomes. In total, 100 µL of the reaction mixture was prepared per vessel. The mixture was incubated for 90 min at 37 °C in darkness, quenching and protein precipitation was carried out by addition of cold MeCN (200 µL, -20 °C). After centrifugation (4 °C, 10 min, 15,000 rpm), 150 µL of supernatant was diluted with 850 µL H_2_O, which was subsequently analyzed by HPLC-qToF-HRMS. The formation of harmane-oxide from 10 µM harmane (1-methyl-9H-pyrido[3,4-b]indole) served as a positive control.

For phase II metabolism, potential sulfation and glucuronidation were investigated. For sulfation, the reaction mixture was pipetted as follows: first, ultrapure water was added and mixed with a 1 mM DTT solution (contains DTT, 100 mM phosphate buffer, 100 µM ascorbic acid, pH 7.4). Then, PAPS (100 µM) and the substance of interest (50 µM) were added, which was concluded by the addition of horse/human cytosol (10 mg protein/mL). For glucuronidation, first, ultrapure H_2_O, phosphate buffer (50 mM) and MgCl_2_ (5 mM) were added to the reaction vessel, followed by addition of the investigated substance (50 µM), UDPGA (5 µM), and horse/human microsomes (1 mg protein/mL), respectively. For both assays, the final volume was 100 µL which was subsequently incubated for 4 h at 37 °C in darkness. Quenching and protein precipitation was achieved by adding 200 µL of cold MeCN (-20 °C). After centrifugation (4 °C, 10 min, 15,000 rpm), 150 µL of supernatant was diluted with 850 µL H_2_O, which was subsequently analyzed by HPLC-qToF-HRMS. For both experiments, 50 µM 7-hydroxycumarin served as positive control.

Chromatographic separation and mass spectrometric detection were identical to the parameters already described for the fragmentation experiments for phase I and phase II glucuronidation metabolism. For sulfation, the HPLC-qToF-HRMS was operated in negative ionization mode at -4.5 kV and otherwise identical source parameters as previously described. Chromatographic separation was achieved on a Nucleodur Gravity C18 (100 x 2 mm, 3 µm) column (Macherey-Nagel, Düren, Germany) by applying the same gradient as previously described (Chapter 2.5).

## 3 Results and discussion

### 3.1 Isolation of acetoxystachybotrydial acetate as reactant 1

In order to obtain higher quantities of phenylspirodrimanes to perform the semi-synthetic modification, large-scale cultivation of *Stachybotrys chartarum* IBT 40288 to isolate and purify ***1*** was conducted as previously described (Steinert et al., 2022), except for a longer cultivation time of 21 days.

### 3.2 Semi-synthetic reaction with l-tryptophan and tryptamine

**Figure 3:**
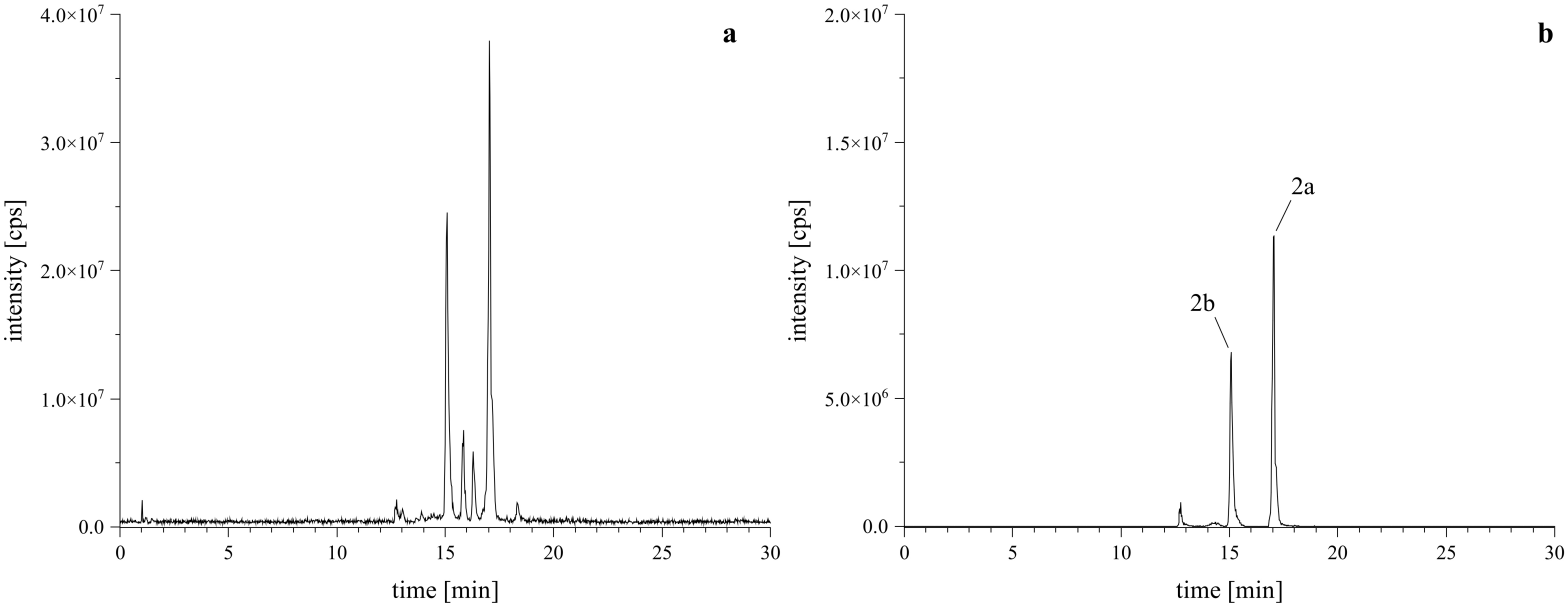
HRMS confirmation of reaction products with l-tryptophan. Left **(A)** is the TIC of the reaction mixture, right **(B)** is the XIC of the [M+H]^+^ calculated as *m/z* 673.3120 ± 2 ppm, confirming the formation of the expected regiospecific isomers (***2a***, ***2b***).

Given that PSDs containing *o*-dialdehyde groups tend to form isobenzofuranones instead of the desired lactam-derivatives, it is essential to maintain mild reaction conditions (McDonald and Sibley, 1981; Steinert et al., 2022). The reaction of PSD with l-tryptophan to obtain acetoxystachybotrylactam acetate-tryptophan (***2***) was performed following the protocol established by our group (Steinert et al., 2022). Unlike our previous findings, this reaction resulted in the formation of two isomeric products (***2a*** and ***2b***), as evidenced by the total ion chromatogram (TIC) and extracted ion chromatogram (XIC) shown in **Figure 3**.

**Figure 4:**
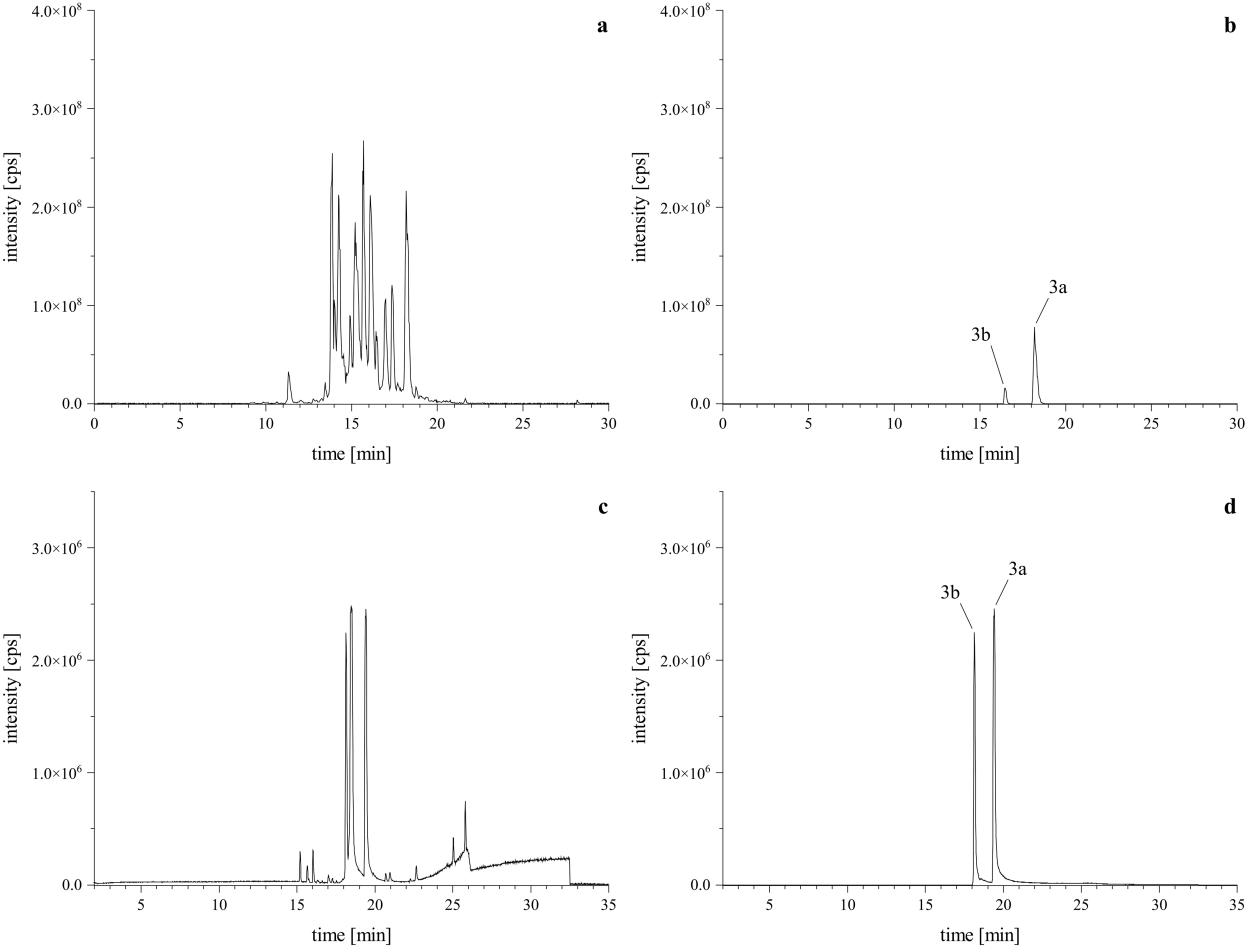
HRMS confirmation of reaction products with tryptamine. Upper left is the TIC of the standard reaction mixture **(A)** *via* HPLC-Orbitrap-HRMS, lower left is the BPC of the optimized reaction mixture **(C)** *via* HPLC-qToF-HRMS. Upper right **(B)** and lower left **(D)** are the XICs of the [M+H]^+^ calculated as *m/z* 629.3226 ± 2 ppm, confirming the formation of the expected regiospecific isomers (***3a***, ***3b***).

Given our previous findings that a lactam-agmatine derivative exhibited the highest activity (Steinert et al., 2022), we aimed to further investigate the impact of the missing carboxyl group from the amino acid. Therefore, we conducted a reaction using tryptamine as a reaction partner under standard conditions. This reaction yielded two regio isomeric products, though in minor amounts (**Figures 4A, B**). To optimize the reaction, we focused on excluding water by employing a molecular sieve and aprotic solvents like DCM, DMF, and DMSO. Additionally, we examined the effect of pH by adding of glacial acetic acid in a half-logarithmic scale (10, 33, 50, and 100 µM). We also assessed the reaction time to determine the optimal conversion rate. Finally, instead of the traditional dropwise addition, we mixed the primary amine (reactant 2) with the PSD (reactant 1) at a controlled flow rate. These optimizations led to a more targeted reaction, favoring the desired products and ensuring the formation of both regio isomeric types, as depicted in the base peak chromatogram (BPC, **Figures 4C, D**).

Since all hypothesized reaction products (***2a***, ***2b***, ***3a***, ***3b***) were successfully matched to the calculated [M+H]^+^ *m/z* values expected for these compounds and showed the previously mentioned regiospecific isomerism, purification of all reaction products was performed.

### 3.3 Purification of reaction products

**Figure 5:**
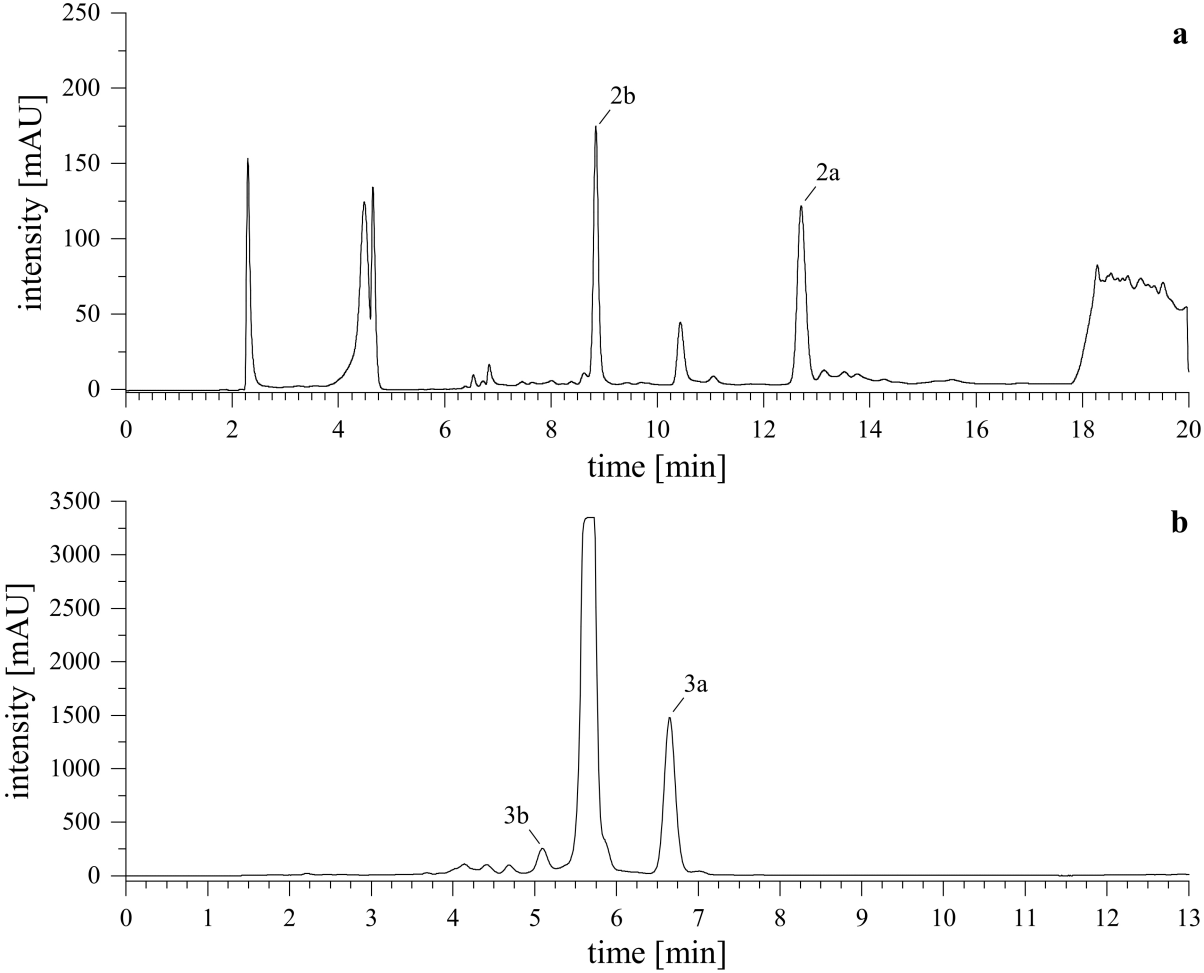
Semi-preparative HPLC-UV chromatograms. Upper **(A)** shows the purification of reaction products from l-tryptophan (***2a***, ***2b***), lower **(B)** shows the purification of reaction products from tryptamine (***3a***, ***3b***). Monitored wavelength was λ = 255 nm.

After lyophilizing the reaction mixture with l-tryptophan (***2a***, ***2b***), the resulting crude extract was reconstituted in MeCN/H_2_O (8/2, v/v) and briefly centrifuged. The mixture was then processed using semi-preparative HPLC-UV. For the derivatives obtained from the reaction with tryptamine (***3a***, ***3b***), DMSO and acid were removed first. Here, a wash-out was conducted by dilution of the reaction mixture to 1% DMSO in water, loading it onto a pre-conditioned C18 SPE cartridge, washing with water, and eluting the reaction products by gradually increasing the organic content of MeCN/H_2_O. The collected fractions were combined, dried *in vacuo* and lyophilized. The resulting crude extract was reconstituted in MeCN and further purified using semi-preparative HPLC-DAD-UV. Representative chromatograms of the purification are shown in **Figure 5**.

After removal of solvent *in vacuo* and subsequent lyophilization, a white powder was obtained. Purity of each substance was determined *via* HPLC-DAD-ELSD by injection of 50 µL of a 100 µg/mL (***2a***, ***2b***) or 50 µg/mL (***3a***, ***3b***) solution in MeCN/H_2_O (8/2, v/v). The compounds ***2a***, ***2b***, and ***3b*** both showed purity of ≥99%, while ***3a*** had a purity of ≥97% (**Supplementary Materials**).

### 3.4 Structure confirmation by NMR and HRMS fragmentation

The structural confirmation of the reaction products focused on verifying the incorporation of the supplied reactant 2 (l-tryptophan or tryptamine) and determining the isomeric identity. The phenylspirodrimane backbone was confirmed in accordance with Jagels et al (Jagels et al., 2019). The signals corresponding to the indole structures were observed at the expected resonances, with δ_H_ = 7.0-8.0 ppm and δ_C_ = 108.8-138.2 ppm, while the amine-group appeared at δ_H_ = 9.1-11.8 ppm. For tryptophan, water signals at δ_H_ = 8.0-9.2 ppm were attributed to the carboxy group, though no δ_C_ could be assigned from HMBC or HSQC experiments, likely due to low concentration of compound. All expected signals for tryptamine were successfully assigned. The connection to the lactam ring of the primary amines was observed as a downfield shift (for tryptophan: δ_H_ = 4.9-5.3 ppm and δ_C_ = 47.2-49.6 ppm; for tryptamine: δ_H_ = 3.8-4.0 ppm and δ_C_ = 43.6-44.4 ppm), which indicated the presence of the electron-withdrawing tertiary nitrogen through its de-shielding effect on neighboring protons and carbons, thereby confirming the linkage of the primary amine through cyclization and lactam formation.

**Figure 6:**
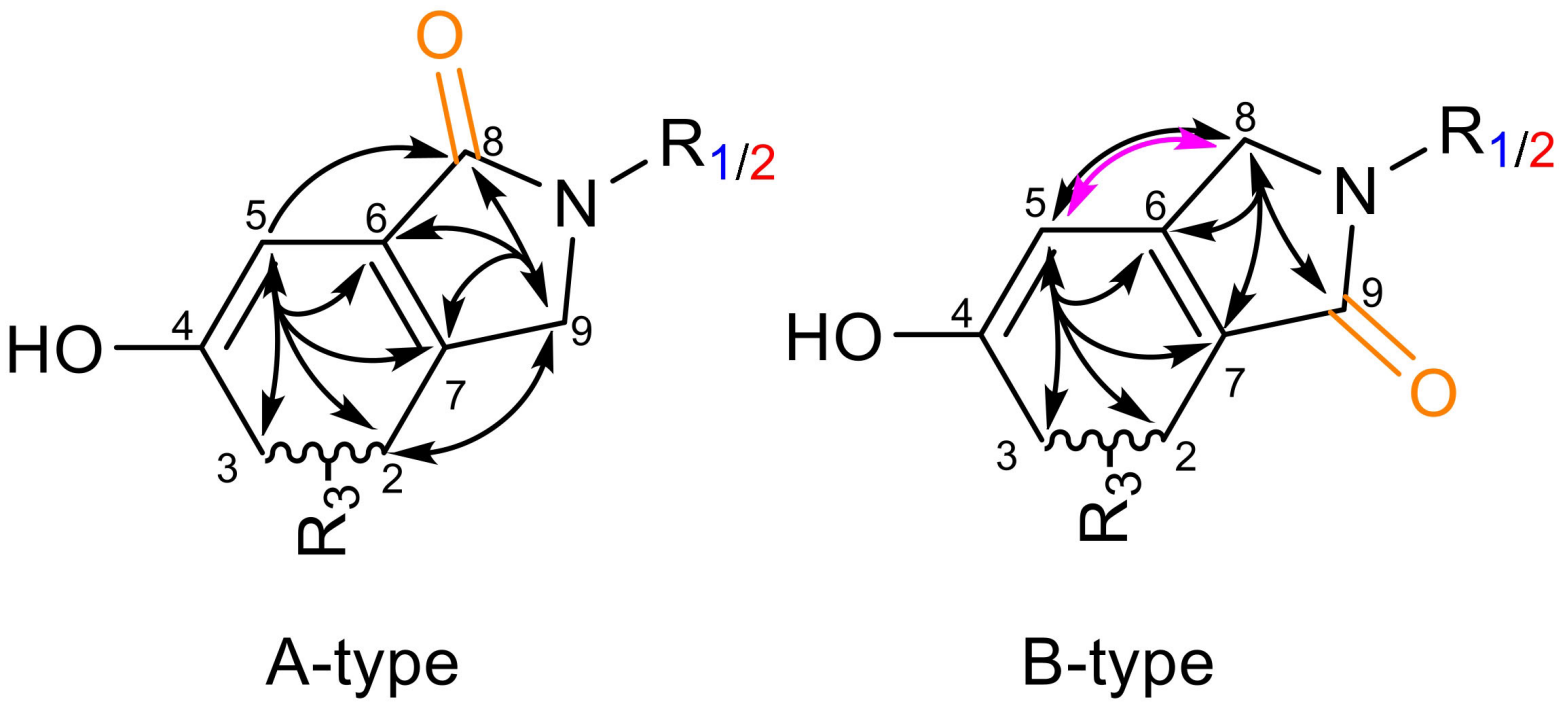
Confirmation of isomeric identity of the reaction products. R_1_ is l-tryptophan, R_2_ is tryptamine, R_3_ is the remaining spirodrimane backbone. A-type is assigned with the carbonyl group at position 8 (orange); B-type has the carbonyl group at position 9 (green). Selected HMBC correlations (black arrows) and specific COSY-correlation (pink arrow) are given.

Regioselective isomerism was evaluated using COSY and HMBC correlations (**Figure 6**). Here, the cyclization and the presence of the novel carbonyl group produced clear ^13^C signals at a down-field shift of δ_C_ = 167.4-171.5 ppm, which was additionally de-shielded by the tertiary nitrogen. In correspondence with the position of the carbonyl group, a down-field shift of the neighboring carbon position was observed, indicating the presence of an electron-withdrawing oxygen. If position 8 (A-type) was connected, position 6 shifted to δ_C_ = 106.2-108.2 ppm, while position 7 shifted to δ_C_ = 114.1-114.6 ppm if the carbonyl was found at position 9 (B-type). The COSY-correlation between the CH_2_ at position 8 and CH at position 5 proved highly specific, as it solely occurred in the B-type isomer. In the A-type isomer, only one HMBC correlation between H5 and C8 was observed. Further of 2D experiments enabled the allocation of HMBC correlations within the newly cyclized isoindolinone.

**Figure 7:**
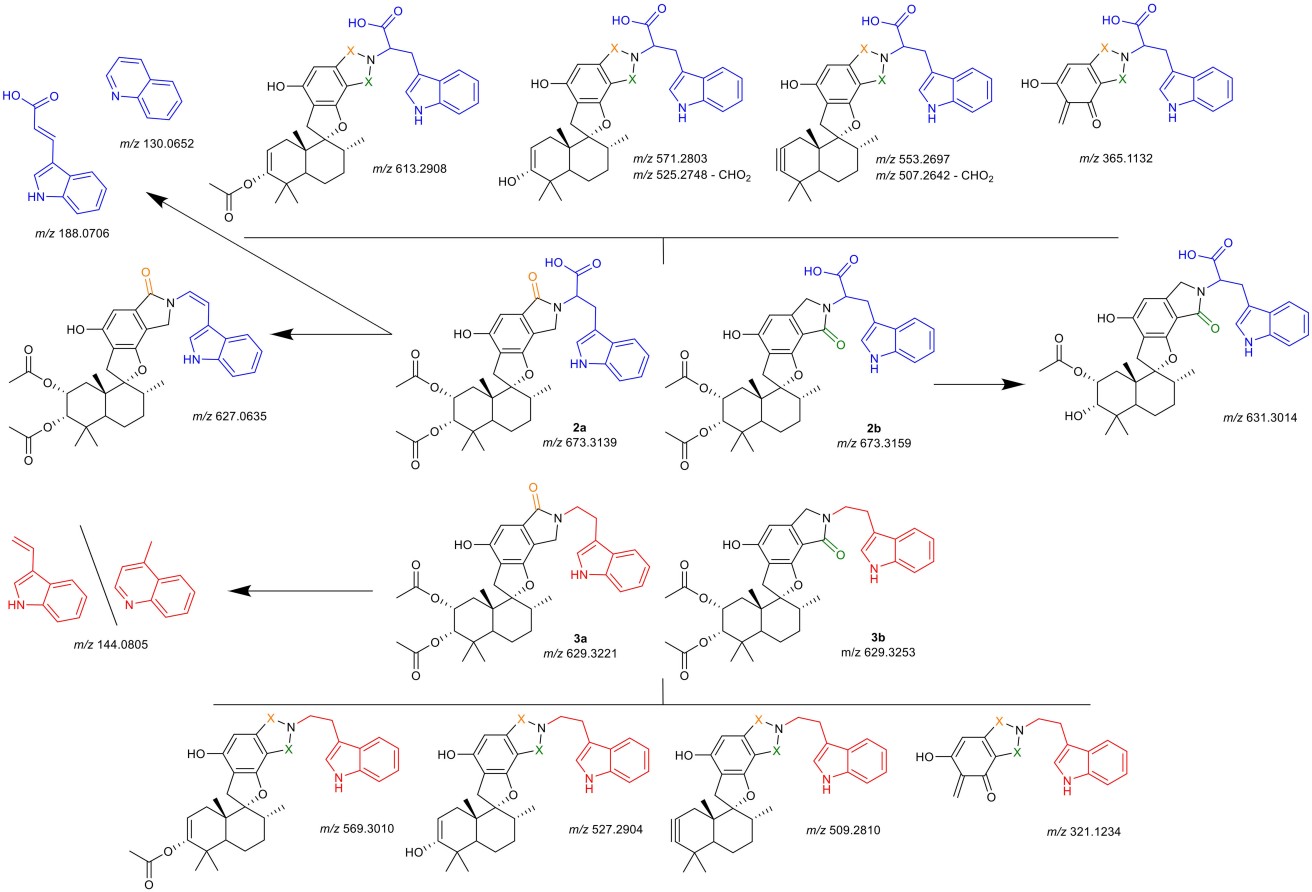
Hypothesized identity of observed fragmentation patterns. Upper half (blue tryptophan-residue) are compounds ***2a*** and ***2b***, lower half (red tryptamine-residue) are compounds ***3a*** and ***3b***. Uppermost and lowermost structures are selected fragments shared by both isomers (indicated by orange or green X); structures indicated with arrows are selected fragments observed solely for the specific isomer, for structures separated by a dash (\) both proposed structures match the *m/z*. Depicted *m/z* values are given as the calculated [M+H]^+^ for the proposed fragment structure, the obtained spectra and mass error calculations are presented in detail in the **Supplementary Materials**.

The isomeric identities of all compounds were successfully established based on the NMR patterns. Compounds ***2a*** and ***3a*** were confirmed as A-type isomers, while ***2b*** and ***3b*** were identified as B-type isomers. Due to the absence of characteristic signals for the carboxy group of ***2a*** and ***2b*** in the 1D and 2D NMR spectra, additional HRMS-fragmentation experiments were performed. These experiments revealed fragmentation patterns specific to each primary amine and its corresponding regio isomer. A summary of the evaluated features is provided in **Figure 7**.

For all compounds, the neutral loss of the acetate groups with formation of either a double- or triple-bond was observed (*m/z* 613.2908, *m/z* 571.2803, *m/z* 553.2697 for ***2a***, ***2b***; *m/z* 529.3010, *m/z* 527.2904, *m/z* 509.2810 for ***3a***, ***3b***). Additionally, a cleavage of the drimane backbone was seen for all reaction products (*m/z* 365.1132 for ***2a***, ***2b***; *m/z* 321.1234 for ***3a***, ***3b***). For both A-type isomers (***2a***, ***3a***) the split of the primary amine was observed as *m/z* 188.0706 (tryptophan) and *m/z* 144.0805 (tryptamine), respectively. For both fragments, a quinolinone-formation is possible, which was observed at *m/z* 130.0652 (tryptophan), while for tryptamine, *m/z* 144.0805 could also correspond to the quinolone. This was a first indication that indeed, compounds ***2a*** and ***2b*** incorporate the carboxy-group that was not observed irrevocably in the NMR experiments. Further verification was noted from the presence of fragments with a neutral cleavage of CHO_2_ together with the formation of a double-bond within the linkage of the indole to the lactam (*m/z* 525.2748, *m/z* 507.2642). For ***2a***, an additional fragment was detected which comprised solely the neutral loss of the carboxy-group (*m/z* 627.0635). This further verified the chemical structures of ***2a***, ***2b***, ***3a***, and ***3b***.

### 3.5 Cytotoxicity and genotoxic potential

**Figure 8:**
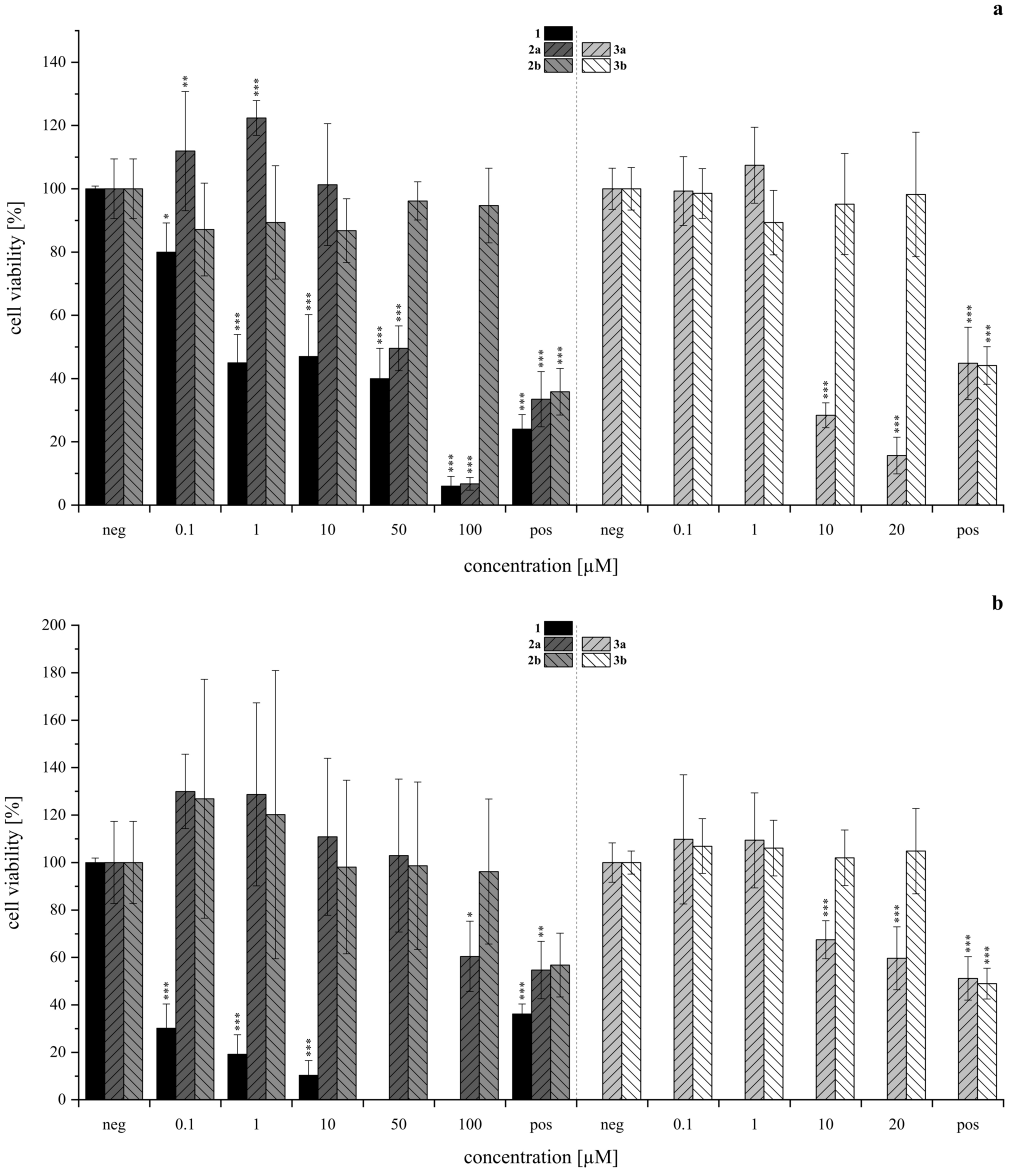
Cytotoxicity of acetoxystachybotrydial acetate (AcAc, ***1***), isoindolinones with l-tryptophan (***2a***, ***2b***, lefthand side) and tryptamine (***3a***, ***3b***, righthand side) assessed *via* the resazurin assay in HepG2 (**A**, upper panel) and A549 (**B**, lower panel) after 24 h of substance incubation normalized to the negative control as T/C (neg, 1% DMSO for *2a*, *2b*; 0.5% DMSO for *3a*, *3b*). Positive control (pos) was 10 µM T-2 toxin. Experiments were conducted with three individual cell passages with three (*2a*, *2b*, *N*≥9) or six (*3a*, *3b*, *N*≥18) biological replicates each. Columns depict mean values ± standard deviations in %. Statistically significance was tested by one-way ANOVA and *post-hoc* Tukey’s test: ^*^p ≤ 0.05, ^**^p ≤ 0.01, ^***^p ≤ 0.001. Data for compound *1* was provided by our group’s previous investigation and has been adjusted accordingly (Steinert et al., 2022).

The cytotoxic and genotoxic potential of the synthesized isoindolinones ***2a***, ***2b***, ***3a***, and ***3b*** was evaluated using the resazurin assay in human liver carcinoma (HepG2, HB-8065, **Figure 8A**) and human lung adenocarcinoma (A549, CCL-185, **Figure 8B**) cells. For compounds ***2a*** and ***2b***, the tested concentration range covered 0.1 µM to 100 µM, with 1% DMSO serving as the negative (neg) control as 1% DMSO was the solvent concentration in each tested dilution of ***2a*** and ***2b***. Due to precipitation issues with compounds ***3a*** and ***3b*** in cell culture medium at higher concentrations, the tested range was limited to 20 µM, with precipitation observed starting at 1 µM for ***3b*** and 10 µM for ***3a***. For these assays 0.5% DMSO was used as negative control as 0.5 % DMSO was the solvent concentration in each tested dilution of ***3a*** and ***3b***. In all assays, 10 µM of T-2 toxin was used as the positive (pos) control. The results of this investigation are depicted in **Figure 8**.

**Figure 9:**
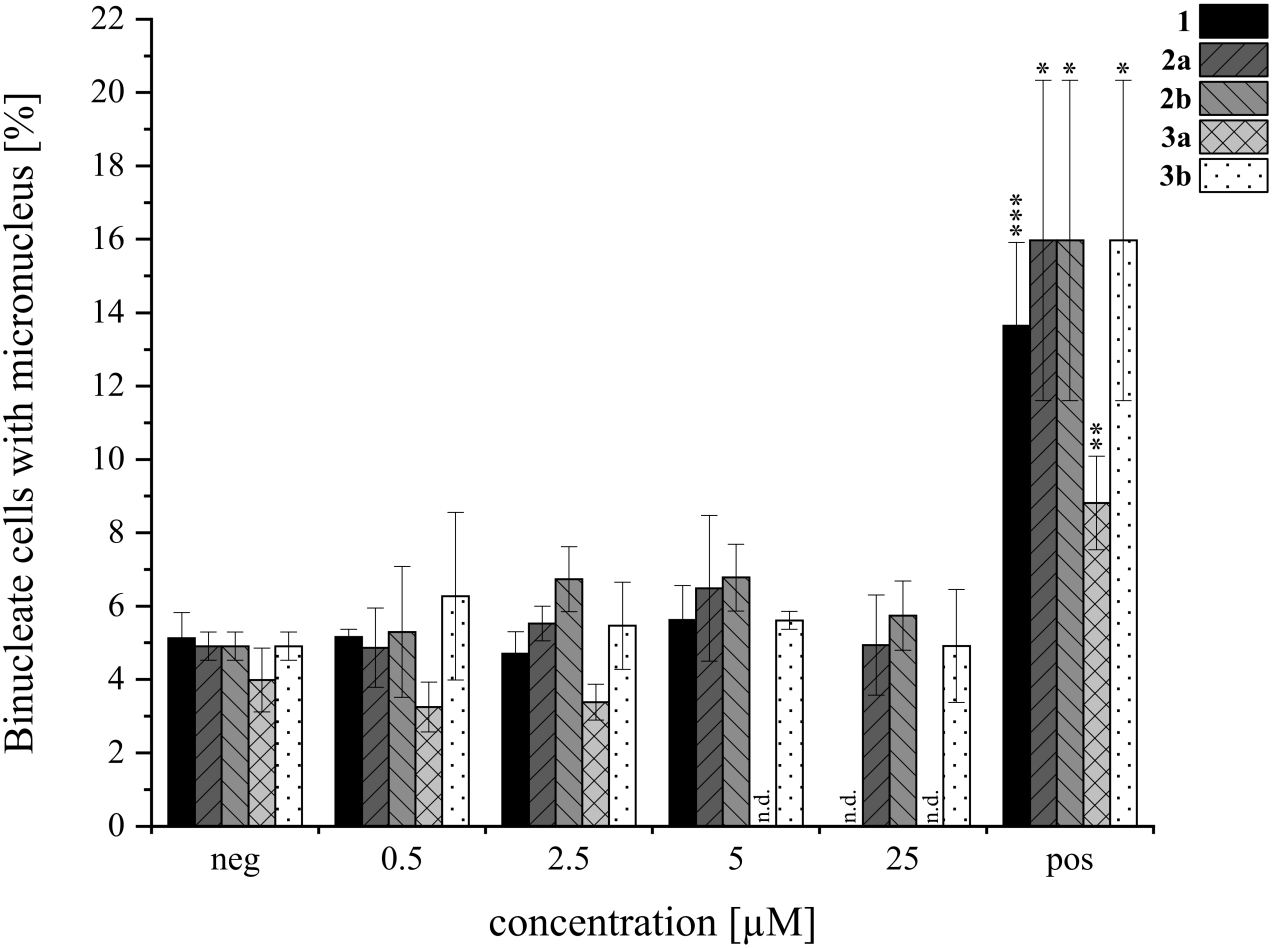
Genotoxicity of AcAc (***1***), its tryptophan-derivatives (***2a***, ***2b***) and tryptamine-isoindolinones (***3a***, ***3b***) in HepG2 *via* the micronucleus assay. Negative (neg) control was 0.5% DMSO, 0.6 µM MMC was the positive (pos) control. Experiments were conducted with three biological replicates each (*N*=3). Columns depict mean values ± standard deviations in %. Statistical significance was tested by student’s T-test: ^*^p ≤ 0.05, ^**^p ≤ 0.01, ^***^p ≤ 0.001, n.d.= not detected.

Overall, the HepG2 (**Figure 8A**) cell line demonstrated greater sensitivity to the tested isoindolinones compared to A549 (**Figure 8B**), with the A-type derivatives (***2a***, ***3a***) showing higher toxicity than the B-type derivatives (***2b***, ***3b***). In the B-type derivatives, the presence (tryptophan) or absence (tryptamine) of the carboxylic acid did not significantly affect cell viability in either cell line across the tested concentration ranges. Despite the precipitation of ***3b***, no impact on cell viability was observed. In contrast, the A-type isomers exhibited pronounced cytotoxic effects, particularly in HepG2 cells. Compound ***2a*** showed similar toxicity to ***1*** at 100 µM (**Figure 8A**, left), ***3a***, lacking the carboxylic acid, was even more toxic than ***1***, starting from 2 µM (**Figure 8A**, right). Similar to ***3b***, also ***3a*** precipitated in DMEM, however, since cell viability was not affected from ***3b***, this was excluded as a cell-death-causing effect. The B-type isomers (***2b***, ***3b***) generally exhibited lower cytotoxic effects, yet the absence of the carboxy group appeared to increase the cytotoxic activity of the tryptamine derivative (***3a***) compared to its tryptophan analog (***2a***). In order to gain further insight into potential modes of toxicity, the genotoxic potential of the four derivatives was investigated *via* the micronucleus assay in HepG2. None of the compounds induced genotoxic damage in concentrations between 0.5 and 25 µM. Yet, the highest concentration of ***1*** (25 µM) left the cells visibly damaged, therefore, these cell culture slides was not evaluated. Consistent with the results of the cytotoxicity assay, compound ***3a*** impaired cell viability even more. Therefore, cells incubated with 5 and 25 µM had to be excluded from evaluation. The results of the micronucleus assay are shown in **Figure 9**.

### 3.6 Biological activity of serine proteases of the blood coagulation cascade and trypsin

**Figure 10:**
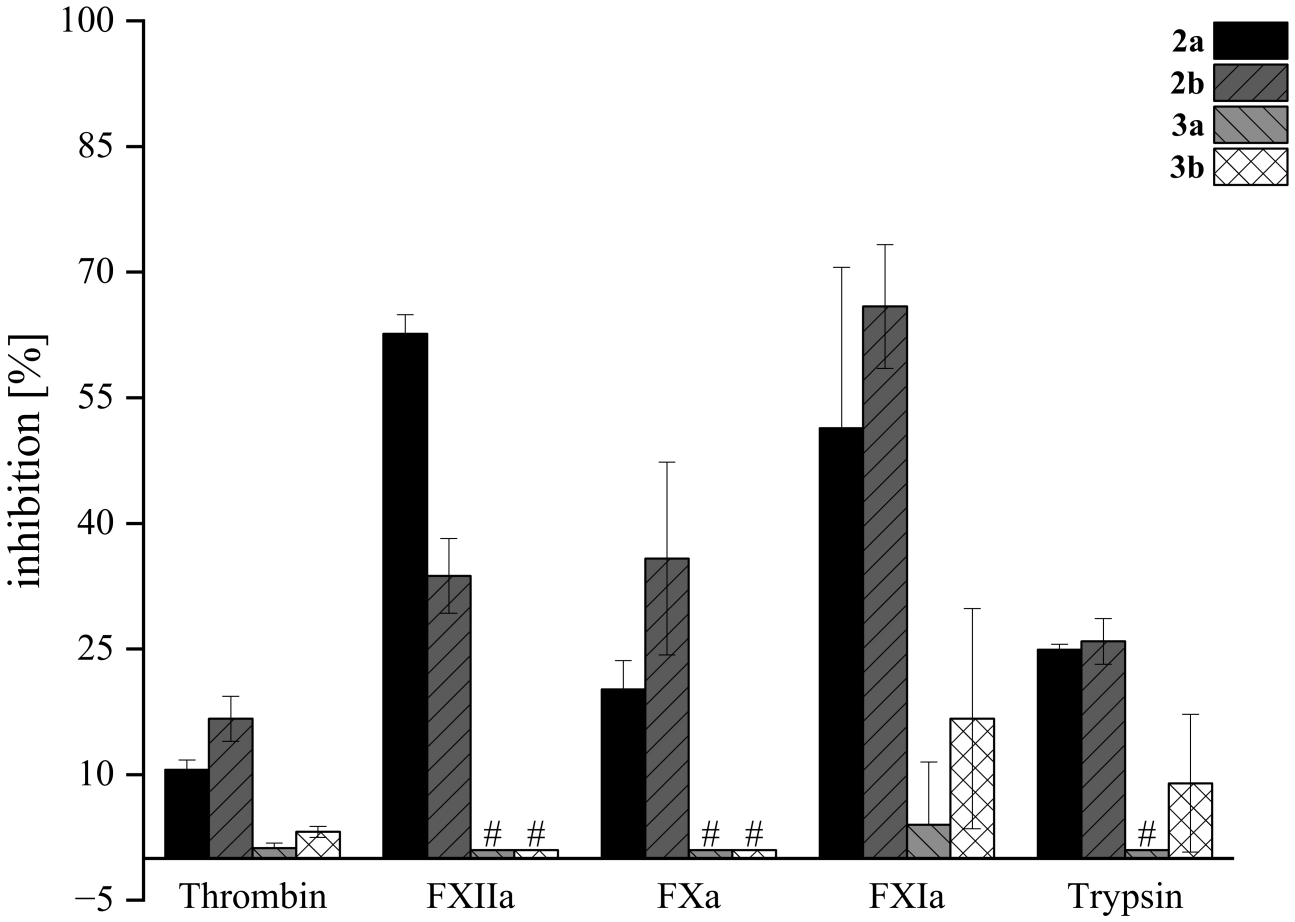
Inhibition of serine proteases by lactams ***2a***, ***2b***, ***3a***, and ***3b***. Compounds were screened at 200 μM in fluorogenic-based assays. Tests were performed in triplicate (*N*=3); mean values ± SD are shown. # For compounds *3a* and *3b*, no inhibition toward FXIIa, FXa, and trypsin was observed. Dabigatran, rivaroxaban, and compound “39b” (Platte et al., 2021) were used as positive controls (not shown) at 500 nM and inhibited thrombin by 98.3 ± 0.1%, FXa by 99.8 ± 0.3%, and FXIIa by 100.2 ± 6.3%, respectively (Steinert et al., 2022). Inhibitor 5t at 8 µM (68.4 ± 0.7%) (Imberg et al., 2022) and inhibitor E at 2 µM (71.2 ± 1.4%) (Erbacher et al., 2024) were used as positive controls (not shown) for FXIa and trypsin, respectively.

As mentioned previously and demonstrated in our earlier study, the tryptophan derivatives of acetoxystachybotrydial acetate ***1*** exhibited the ability to inhibit thrombin by 50% during micro-scale screening (Steinert et al., 2022). Consequently, individual compounds of L-tryptophan- and tryptamine-derived lactams, and their isomeric forms were tested against selected serine proteases involved in the blood coagulation cascade, including thrombin, factor XIIa, FXa, and FXIa, as well as against trypsin, which served as a specificity control. Interestingly, both isomeric forms of the tryptophan-derived lactams (***2a*** and ***2b***) showed greater activity than the tryptamine derivatives (***3a*** and ***3b***) against all tested serine proteases (**Figure 10**). This is in opposite to our previous observation, where agmatine derivatives lacking the carboxylic acid moiety showed superior inhibitory activity compared to arginine derivatives, exhibiting the carboxylate residue Steinert et al. (2022). This might be associated with a lower flexibility of bulky tryptophan/tryptamine derivatives compared to arginine/agmatine derivatives. The lower flexibility might lead to lower ability to adapt an active conformation in the active site of the enzymes.

The highest inhibitory activity was observed for compounds ***2a*** (51%) and ***2b*** (66%) when tested against FXIa. In most experiments, the B-type isomer of the tryptophan derivatives exhibited higher activity compared to the A-type isomer, except in the case of FXIIa, where the A isomer was more active (63%) than the B form (34%). Notably, tryptamine derivatives of acetoxystachybotrydial acetate (***3a*** and ***3b***) demonstrated low or negligible activity, with the highest inhibition observed for FXIa at 17% and 9% for ***3b*** and ***3a***, respectively. At our screening conditions tryptophan-derived lactams ***2a*** and ***2b*** exhibited a stronger inhibitory effect on the intrinsic blood coagulation pathway enzymes FXIa and FXIIa compared to thrombin and FXa, which are part of the common pathway. Targeting enzymes of the intrinsic pathway, such as FXIa and FXIIa, represents a promising approach for the development of safer anticoagulant drugs. This strategy is gaining attention because these enzymes play a crucial role in thrombosis, while their inhibition is associated with a reduced risk of bleeding complications, making them attractive targets for new antithrombotic therapies (Al-Horani and Desai, 2016; Kalinin, 2021). While tryptophan derivatives showed moderate activity against physiologically relevant serine proteases, their activity was not higher than that of the previously discovered agmatine derivative.

### 3.7 Hepatic metabolism studies

**Figure 11:**
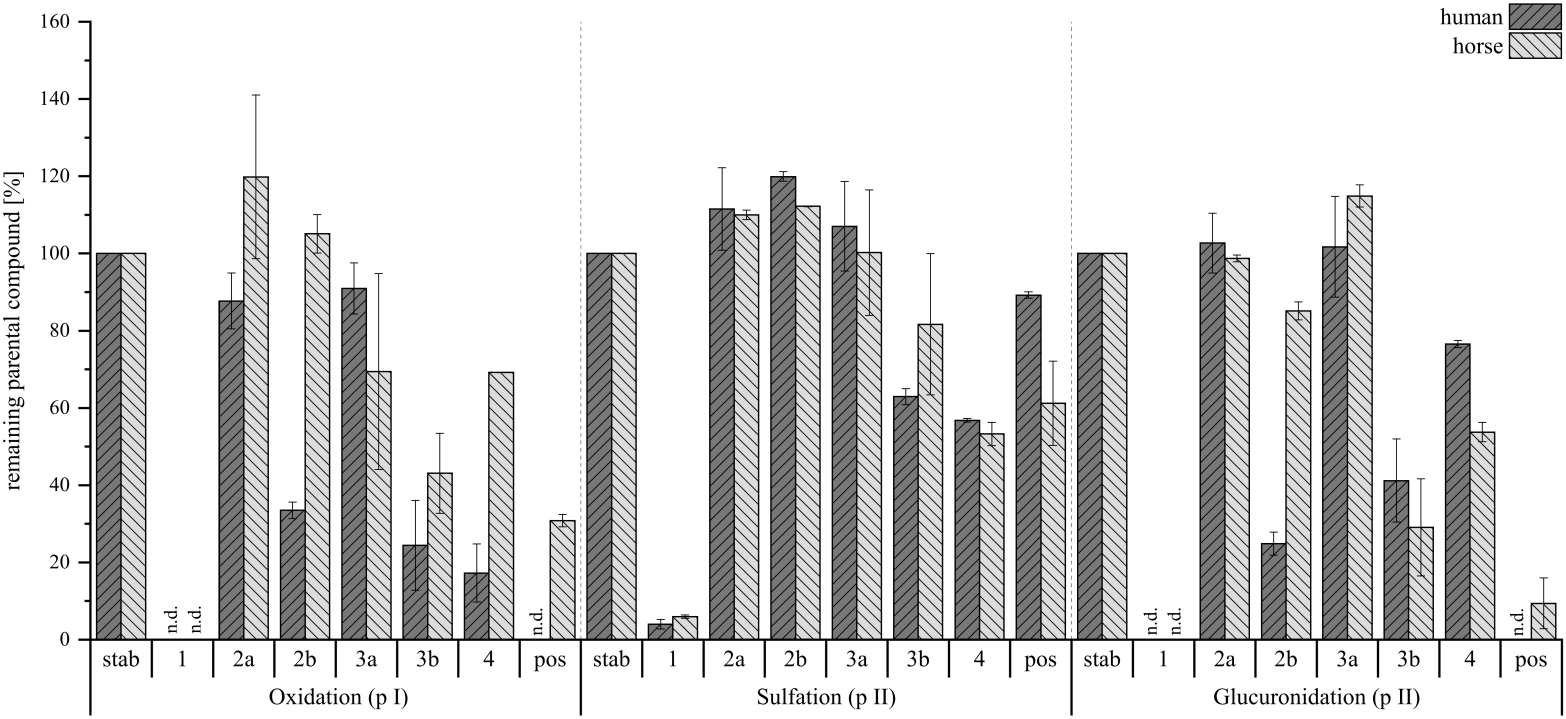
Percentage of remaining parental compound in the reaction solution normalized to the stability control (stab) without microsomes or cytosol. Dark gray bars show human hepatic metabolism, light gray bars depict horse hepatic metabolism. Left is oxidation (phase I, pI), middle is sulfation (phase II, pII) and right is glucuronidation (phase II, pII). Positive controls (pos) are harmane for phase I and 7-hydroxycoumarin for both phase II reactions. Investigated compounds were AcAc (***1***) and its regiospecific isomeric derivatives with tryptophan (***2a***, ***2b***), tryptamine (***3a***, ***3b***), and agmatine (***4***). Stability controls were prepared with an *N*=1, compounds were tested with *N*=2 per species. Depicted are mean values ± range in %, n.d., not detected.

Hepatic metabolism studies are crucial for gaining a deeper understanding of a compound’s biotransformation. In this study, the isoindolinones (***2a***, ***2b***, ***3a***, ***3b***) and acetoxystachybotrydial acetate (***1***) were subjected to phase I (oxidation and reduction) and phase II (sulfation and glucuronidation) metabolic processes. Giving the previous findings by our group, which highlighted the anticoagulant activity of acetoxystachybotrylactam acetate-agmatine (***4***) – an A-type derivative formed from the reaction of AcAc and agmatine (Steinert et al., 2022) – this compound was also included in the study. Besides human exposure to *Stachybotrys*’ toxins, an increased sensitivity of horses toward these secondary metabolites has been reported (Drobotko, 1945), therefore, both human and horse hepatic metabolism was simulated using microsomes and cytosol in accordance to Lindemann et al (Lindemann et al., 2022). The rate of metabolic conversion of each compound was assessed by measuring the remaining amount of the non-metabolized substance in the reactant solution. The findings of this investigation are presented in **Figure 11**.

For the phase I metabolism study, oxidation and reduction were examined at 10 µM compound concentration. Here, a conversion was observed for all tested compounds, with compound ***1*** being metabolized fastest. The semi-synthetic derivatives (***2a***, ***2b***, ***3a***, ***3b***, ***4***) also showed conversion, with the B-type isomers being more susceptible to phase I reactions. Notably, ***4*** was the fastest among the reaction products to be metabolized.

**Figure 12:**
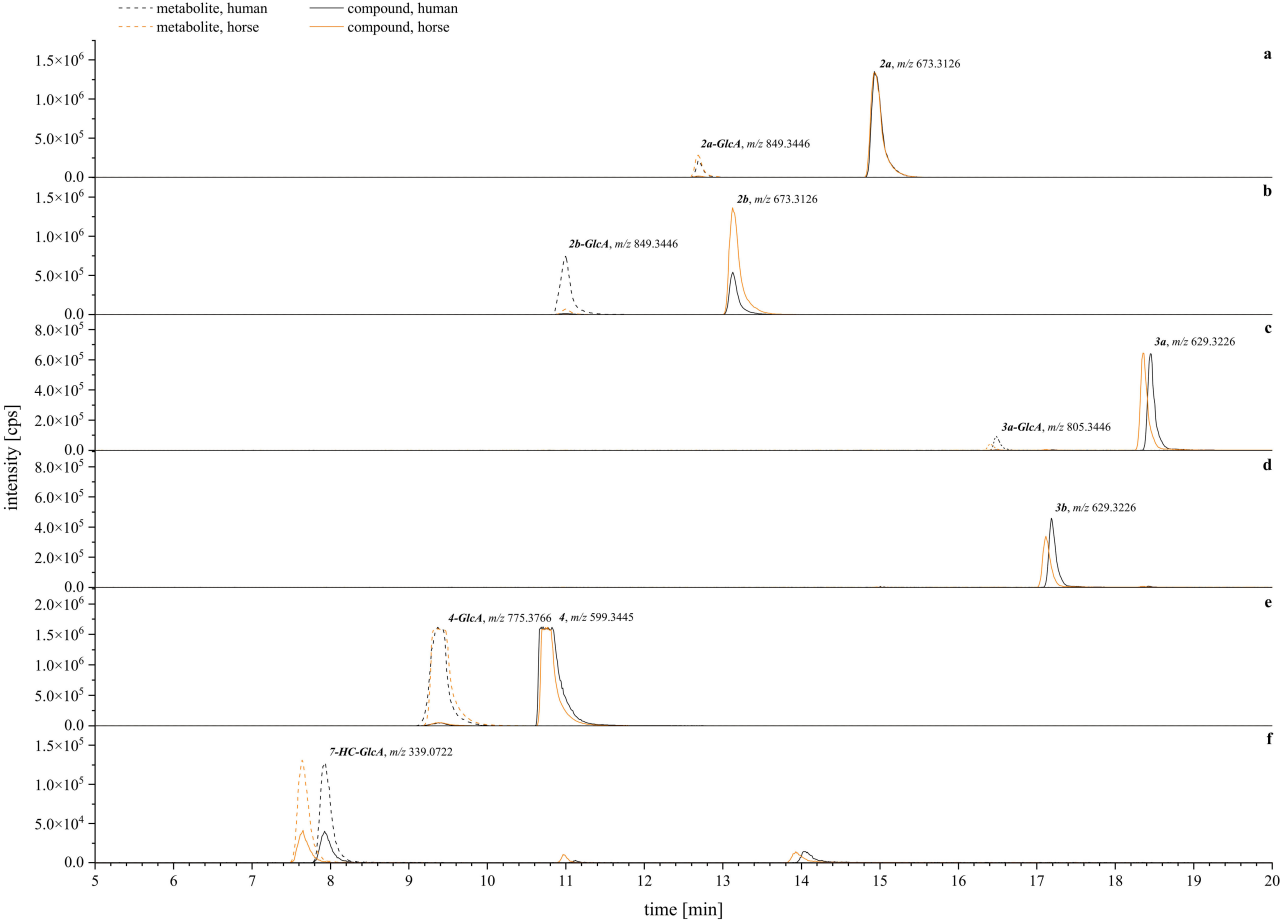
Exemplary XIC chromatograms of the expected glucuronides [M+GlcA+H]^+^ generated from the calculated *m/z* ± 10 ppm. Compounds are assigned with their glucuronide (GlcA) as follows: **(A)** = ***2a*** (tryptophan, A-type), **(B)** = ***2b*** (tryptophan, B-type), **(C)** = ***3a*** (tryptamine, A-type), **(D)** = ***3b*** (tryptamine, B-type), **(E)** = ***4*** (agmatine, A-type), **(F)** = positive control (7-hydroxycoumarin, 7-HC). Human metabolism is given in black lines, horse metabolism is given in orange lines. Solid lines are for parental compounds, dashed lines depict the glucuronized metabolites. A table with the screened metabolites, detected *m/z*, and mass error is given in the **Supplementary Materials**.

Next, sulfation and glucuronidation were studied to assess the phase II hepatic conversion at 50 µM of compound concentration. The tested compounds exhibited a lower tendency for sulfation, as higher levels of parent compounds remained in the reaction solution compared to glucuronidation. While compounds ***1***, ***3b***, and ***4*** did undergo metabolism in both human and horse hepatic cytosol, no sulfated metabolites were detected for any of the substances. Conversely, glucuronidation followed a pattern similar to that observed in phase I metabolism, with no ***1*** remaining in solution and the B-type reaction products (***2b***, ***3b***) more readily converted compared to the A-type (***2a***, ***3a***). Again, ***4*** was the exception, as it showed less susceptibility toward the addition of a glucuronide. Despite this, all reaction products formed glucuronides, though ***3b*** did so minimally, and compound ***1*** did not at all. This suggests that alternative side reactions may have occurred, potentially involving the free *o*-dialdehyde groups of compound ***1***. Exemplary XIC chromatograms of the expected glucuronides are presented in **Figure 12**. The corresponding HRMS-spectra are given in the **Supplementary Materials**.

Closer examination of the phase II glucuronidation showed that most of the detected metabolites corresponded to the conversion rate assessment. From the tryptophan-derivatives, ***2b*** was more available for glucuronidation than ***2a***, and more readily so for human compared to horse hepatic metabolism (**Figures 12A, B**). In contrast, the tryptamine-derivatives were generally less likely to undergo phase II glucuronidation (**Figure 12C, D**). Surprisingly, for compound ***3b***, lower amounts of glucuronized metabolites were detected compared to its corresponding A-type isomer (***3a***), although it was metabolized faster (**Figure 11**). Overall, the best conversion in phase II glucuronidation was observed for compound ***4*** with no species difference (**Figure 12E**) as indicated by the similar signal intensities. Regarding the overall species differences, all tested systems showed a tendency toward a lower conversion rate in horse to human hepatic metabolism (**Figures 11**, **12**). Comparable conversion rates were achieved for compound ***1*** and the sulfation experiments. The horse metabolism was slightly faster during the glucuronidation of ***3b*** and ***4***.

Overall, these results align well with the cytotoxic profiles of the compounds (**Figure 8**). The less cytotoxic derivatives (***2b***, ***3b***) toward human liver carcinoma (HepG2) cells were metabolized more quickly (**Figure 11**) whereas the more toxic substances (***2a***, ***3a***) were metabolized less readily. The case of compound ***1*** and its agmatine-derivative (***4***) is particularly intriguing. Compound ***1*** only showed non-metabolized remnants after phase II sulfation (**Figure 11**) at 50 µM, yet this concentration did decrease HepG2 cell viability already below 50 % (**Figure 8**). This might be due to an interaction between the compound and buffer or medium components. Our group has previously demonstrated the broad activity reactivity of ***1***’s *o*-dialdehyde groups in miniature-scale reactions (Steinert et al., 2022). Considering the complexity of e.g., cell culture medium, it is possible that ***1*** reacted with any available nucleophile to form other semi-synthetic products. These could then either undergo detoxification *via* cellular metabolism or exert cytotoxic effects. Regarding compound ***4***, previous research by our group identified this agmatine derivative as an A-type isomer (Steinert et al., 2022). Interestingly, compound ***4*** exhibited no cytotoxicity in HepG2 and A549 cells, and its activity in the blood coagulation cascade was superior to that of the semi-synthetic products ***2a***, ***2b***, ***3a***, and ***3b*** presented here. This suggests that the observed differences in cytotoxicity, activity, and metabolism between A- and B-type isomers are specific to the comparison between tryptophan and tryptamine derivatives, underscoring the significance of the primary amine used.

## Data Availability

The datasets presented in this study can be found in online repositories. The names of the repository/repositories and accession number(s) can be found in the article/**Supplementary Material**.
