# Supplementary material for: Biological evaluation of semi-synthetic isoindolinone isomers produced by Stachybotrys chartarum
Source: Front Fungal Biol. 2024 Nov 22;5:1494795. doi: 10.3389/ffunb.2024.1494795 (PMC11621054; doi:10.3389/ffunb.2024.1494795)
Supplement: Supplementary file 1 [file DataSheet1.pdf]

Supplementary Material

Biological evaluation of semi-synthetic isoindolinone isomers produced by *Stachybotrys chartarum*

Alica Fischle[1,2], Ulrich Schreiber[1], Viola Haupt[1], Felix Schimang[1], Lina Schürmann[1], Matthias Behrens[1], Florian Hübner[1], Melanie Esselen[1], Dmitrii V. Kalinin[3], Svetlana A. Kalinina[1,2*]

[1]Instititue of Food Chemistry, University of Münster, Corrensstraße 45, 48149 Münster, Germany

[2]Graduate School of Natural Products, University of Münster, Corrensstraße 43, 48149 Münster, Germany

[3]Institute of Pharmaceutical and Medicinal Chemistry, University of Münster, Corrensstraße 48, 48149 Münster, Germany

*** Correspondence:**
Svetlana A. Kalinina
s_kali03@uni-muenster.de

1 Analytical data of reaction products

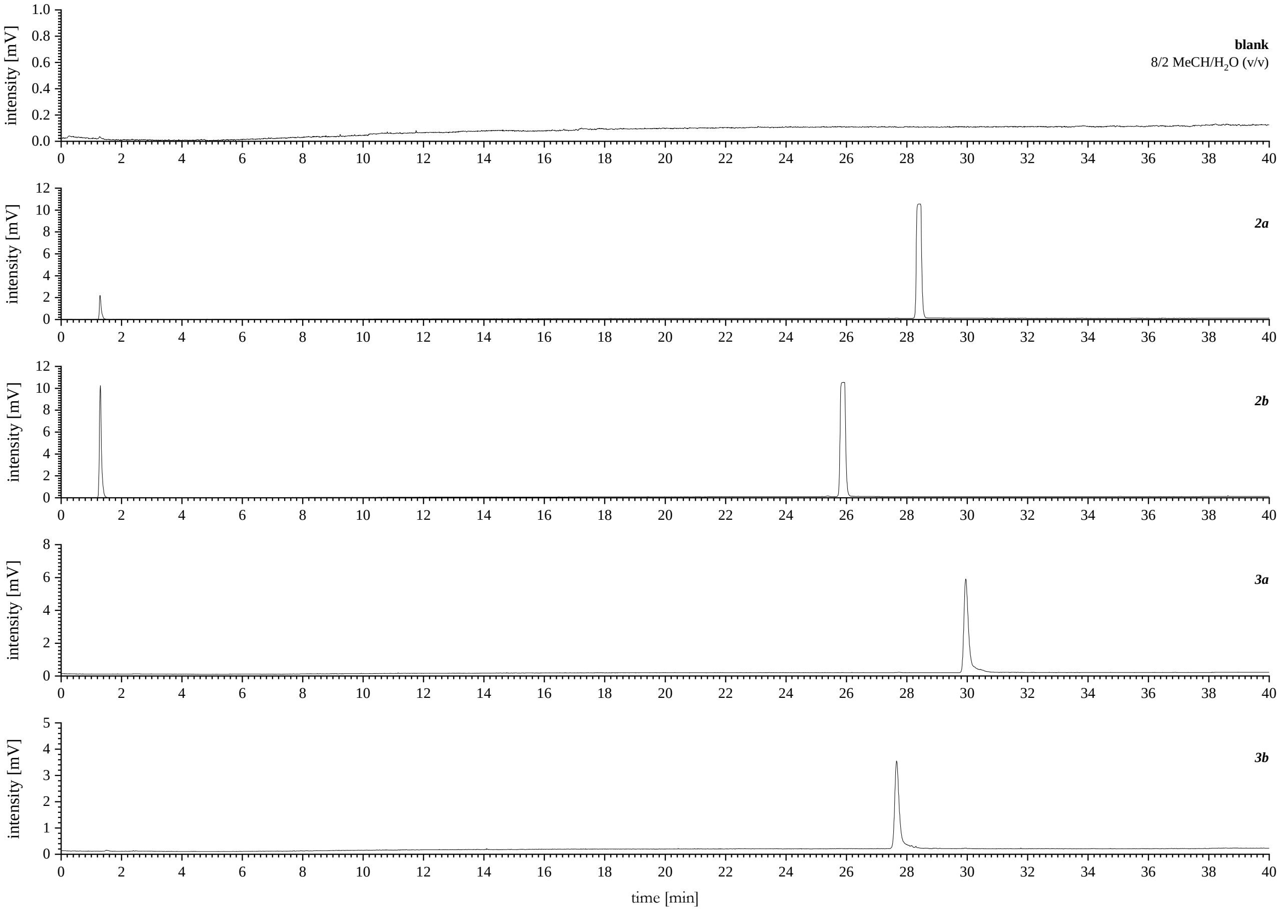

Figure S1. Purity chromatograms of semi-synthetic products. Chromatographic separation was achieved with MeCN/H_2O + 0.1% FA on a Phenyl-Hexyl (250 x 4 mm, 5 µm) column equipped with a guard column of the same material (4 x 3 mm) using a binary gradient increasing from 5-95% organic phase. ELSD parameters were at 350 kPa of compressed air, 10 gain, and 50 °C. Prior to injection, a blank of 8/2 MeCN/H_2O (v/v) was measured. Compounds ***2a*** and ***2b*** were injected as 100 µg/mL solutions, retained at 28.4 min and 25.8 min, respectively, and showed purity ≥99%. Compound ***3a*** and ***3b*** were injected as 50 µg/mL solutions, retained at 30.0 min and 27.7 min, and showed purity ≥99% and ≥97%, respectively.

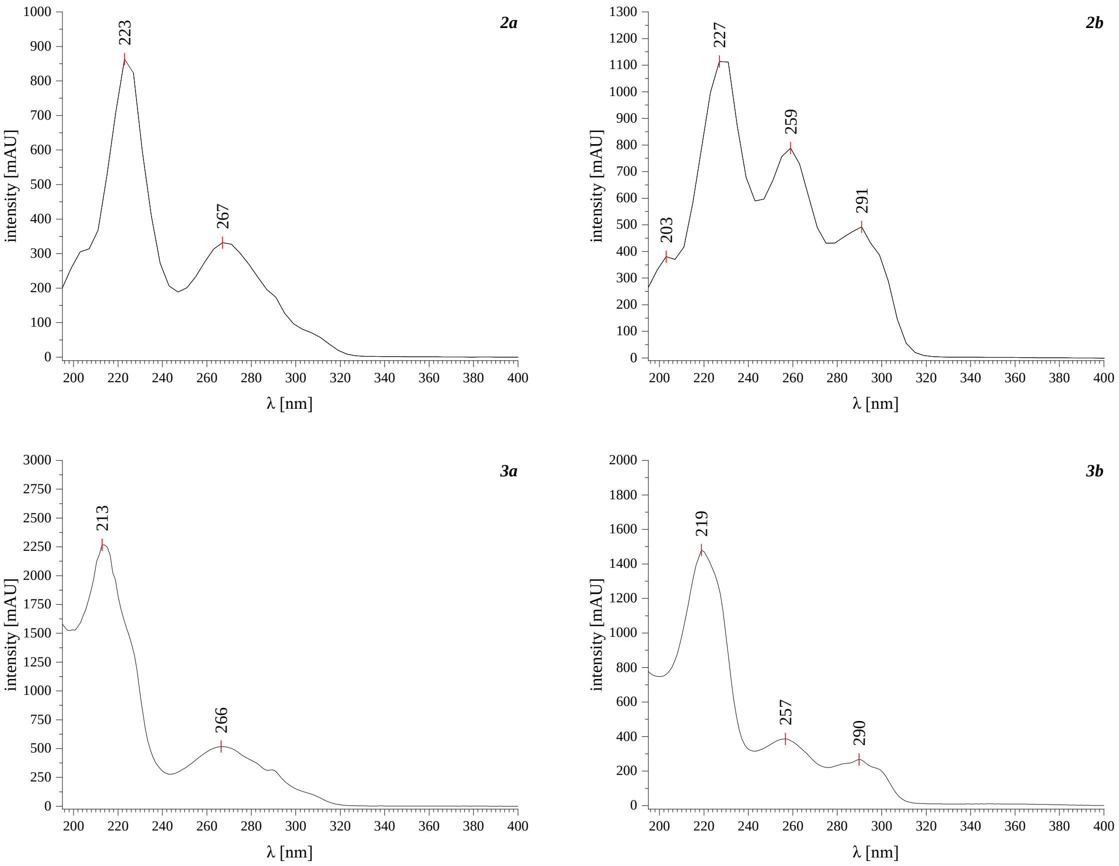

Figure S2. UV-spectra of purified semi-synthetic products. UV-maxima were recorded during chromatographic separation with MeCN/H_2O + 0.1% FA on a phenyl-hexyl (250 x 4 mm, 5 µm) equipped with a guard column of the same material (4 x 3 mm) using a binary gradient increasing from 5-95% organic phase. Wavelength were monitored from 195-650 nm. Compound ***2a*** had λ_{max} = 223, 267; compound ***2b*** had λ_{max} = 203, 227, 259, 291; compound ***3a*** had λ_{max} = 213, 266; compound ***3b*** had λ_{max} = 219, 257, 290.

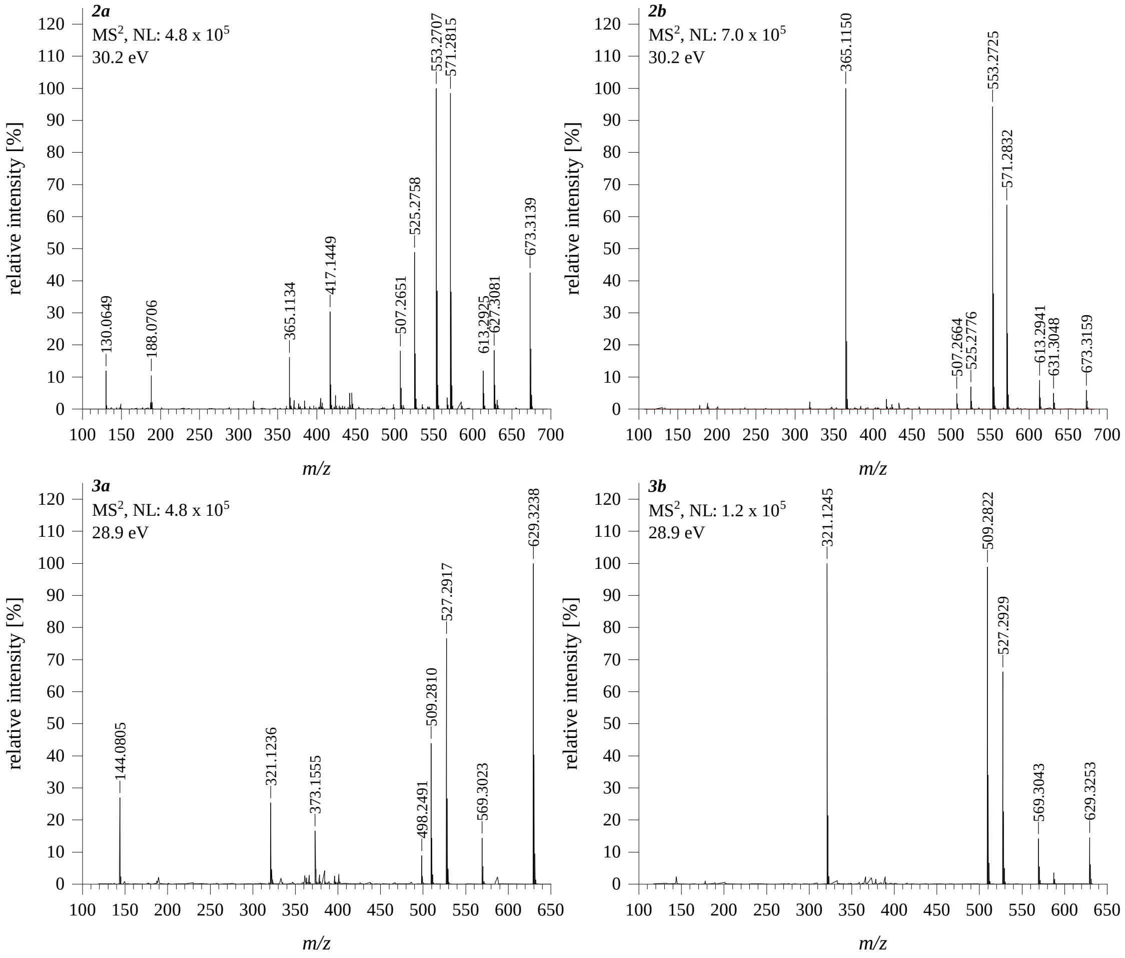

Figure S3. MS^2 spectra of purified semi-synthetic products. Prior chromatographic separation occurred on a ReprosilGold C18 (150 x 2 mm, 3 µm) column equipped with a guard column of the same material (5 x 2 mm) with a binary gradient of MeCN/H_2O + 0.1% FA from 10-100% organic phase. Detection occurred with a HR-ESI-qTOF-MS in positive ionization mode at 4.5 kV capillary voltage and 0.5 kV end plate offset. Nebulizer gas was 2.0 bar, dry gas flow was 10 L/min. Fragmentation occurred in Auto-MS/MS mode selecting 3 precursors and exclusion after 3 spectra. Absolut threshold (1,000 sum) was 48 cts, applied collision energy is given for each compound specifically. Obtained MS^2 spectra were normalized to the most intense *m/z* (NL: normalization level) to give the relative intensity in %. Compounds ***2a*** and ***2b*** were fragmented at 30.2 eV, compounds ***3a*** and ***3b*** fragmented at 28.9 eV.

Table S1. Overview of the MS^2 fragments of semi-synthesized compounds. Detection occurred with a HR-ESI-qToF-MS in positive ionization mode. Shown are the *m/z* values of the fragmented parent ion's proton adduct [M+H] with the mass error (Δm) in ppm, collision energy (CE) applied in eV, the detected fragment *m/z* and the Δm of each fragment ion.

Compound	Parent ion *m/z* $[M+H]^+$ ± Δm	CE [eV]	Fragment *m/z* $[M+H]^+$	Δm [ppm]
2a	673.3139 ± 1.9	30.2	627.3081	-2.5
			613.2925	-2.8
			571.2815	-2.1
			553.2707	-1.8
			525.2758	-1.9
			507.2651	-1.8
			417.1449	-1.0
			365.1134	-0.6
			188.0706	0.00
			130.0649	2.3
2b	673.3159 ± 4.9	30.2	631.3048	-5.4
			613.2941	-5.4
			571.2832	-5.1
			553.2725	-5.1
			525.2776	-5.3
			507.2664	-4.3
			365.1150	-4.9
3a	629.3238 ± 1.9	28.9	569.3023	-2.3
			527.2917	-2.5
			509.2810	-2.2
			498.2491	-1.0
			373.1555	-2.1
			321.1236	-0.6
			144.0805	2.1
3b	629.3253 ± 4.3	28.9	569.3043	-5.8
			527.2929	-4.7
			509.2822	-4.5
			321.1245	-3.4

2 Acetoxy stachybotrylactam acetate-tryptophan 2 (*2a*)

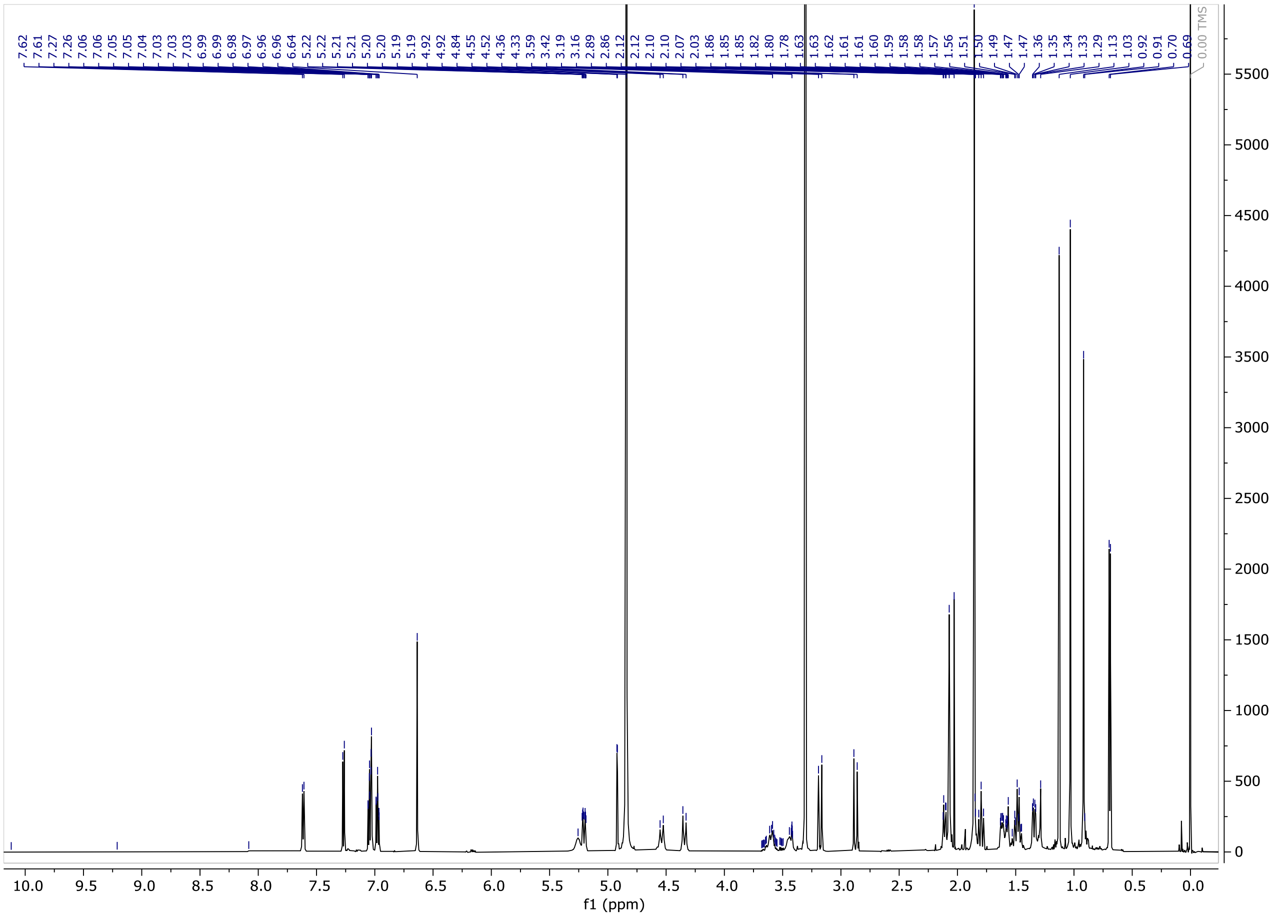

Figure S4. ^{1}H spectrum of ***2a*** in CD_3OD at 600 MHz.

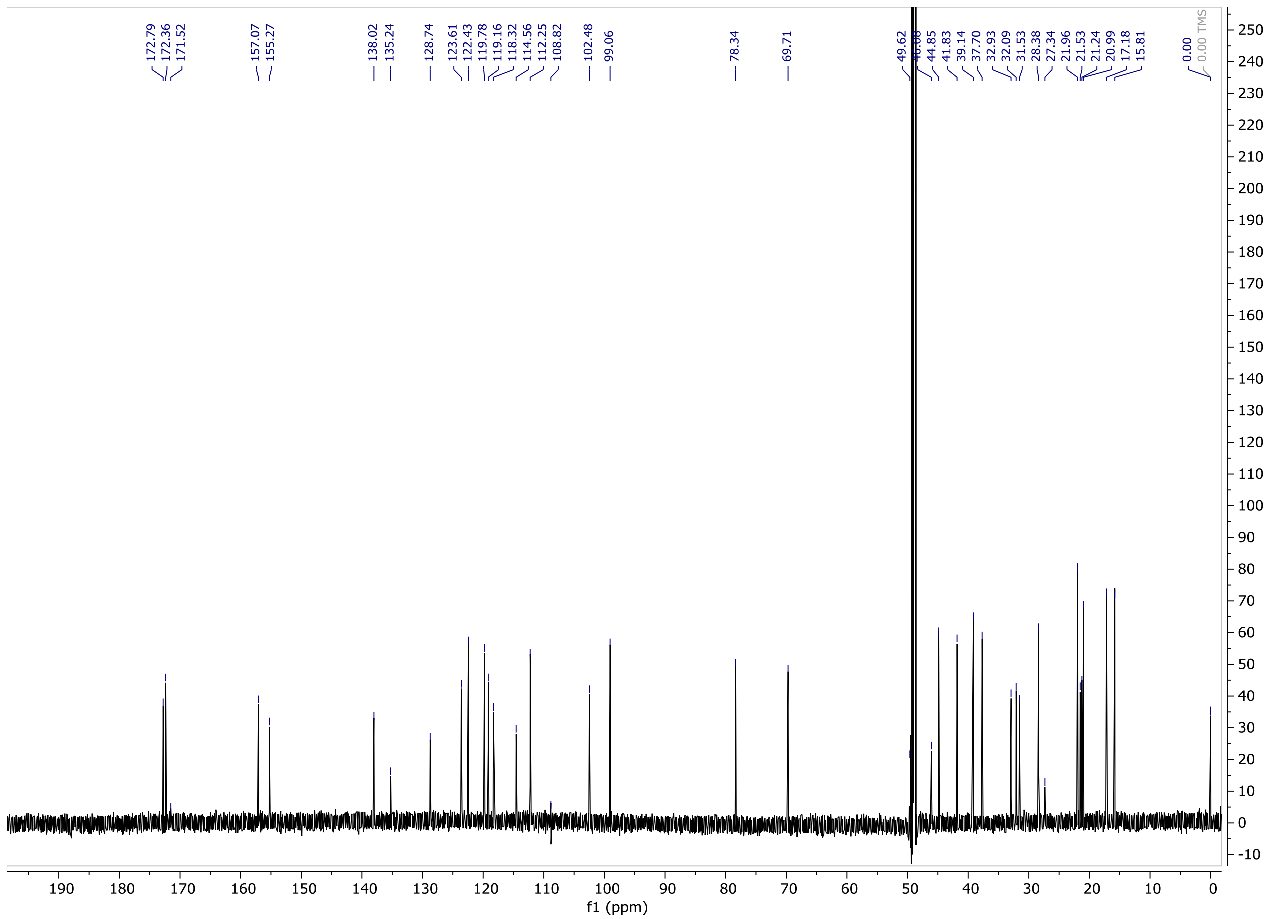

Figure S5. ^{13}C spectrum of ***2a*** in CD_3OD at 150 MHz.

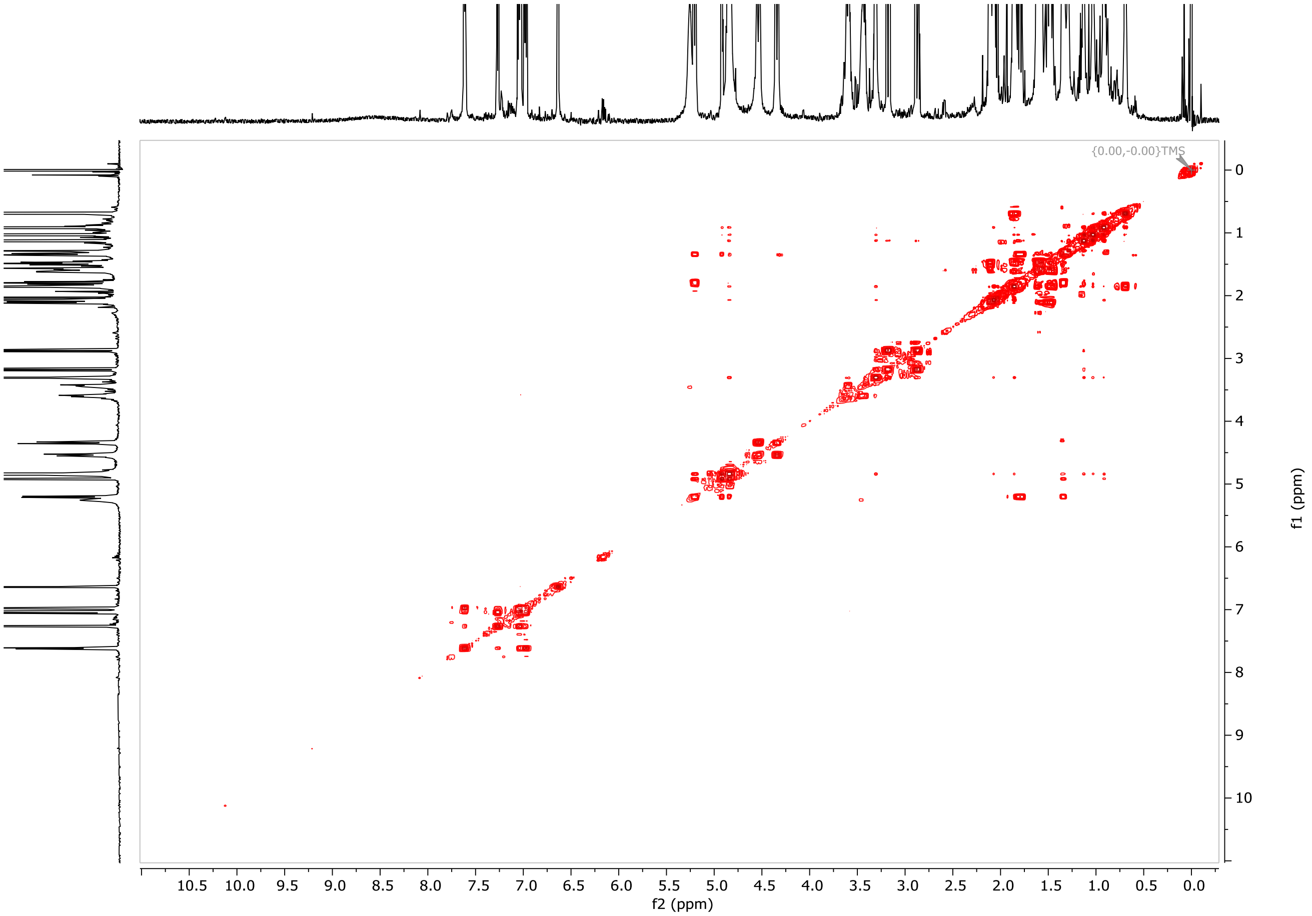

Figure S6. COSY spectrum of ***2a*** in CD_3OD at 600 MHz.

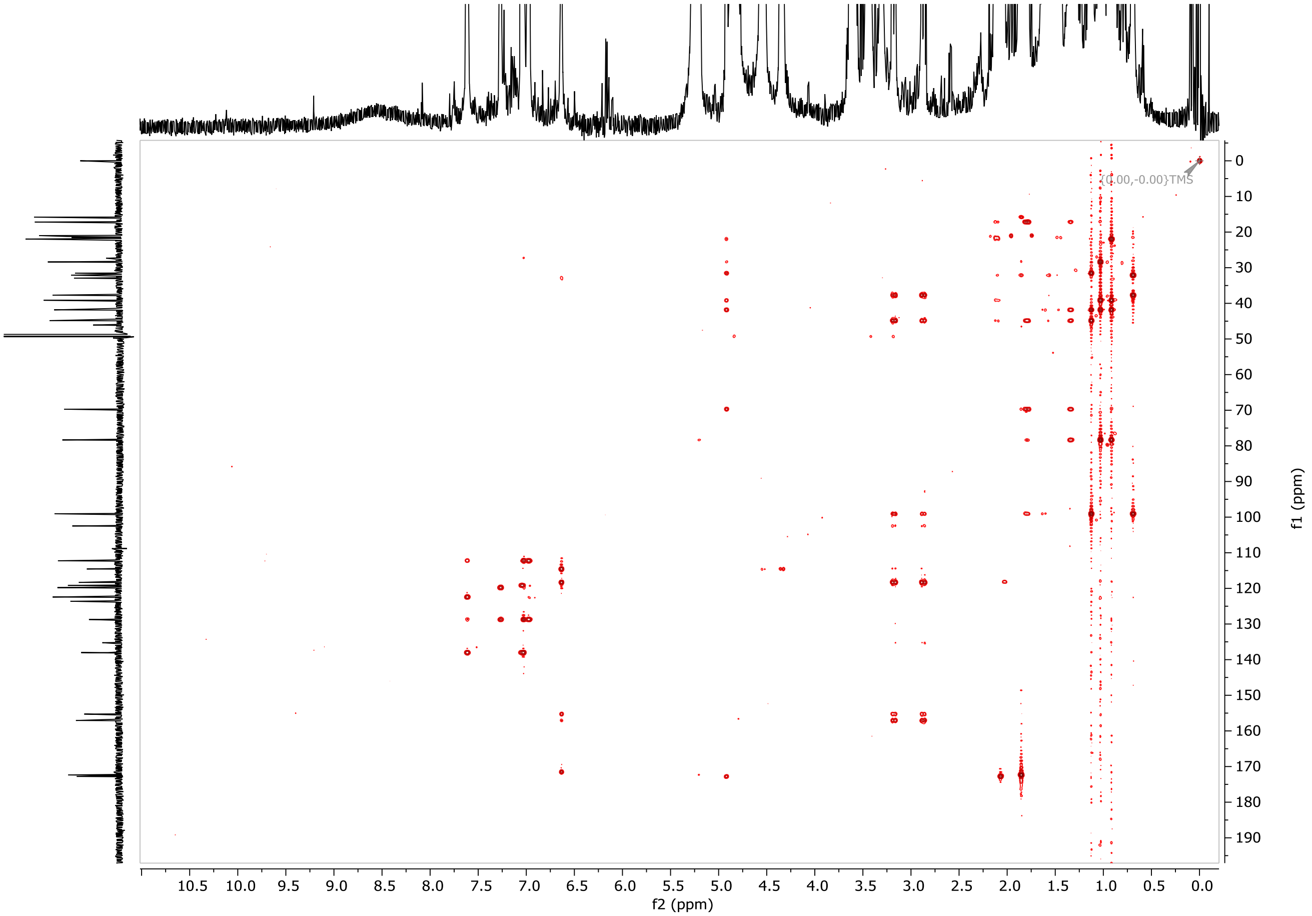

Figure S7. HMBC spectrum of ***2a*** in CD_3OD at 150 MHz and 600 MHz.

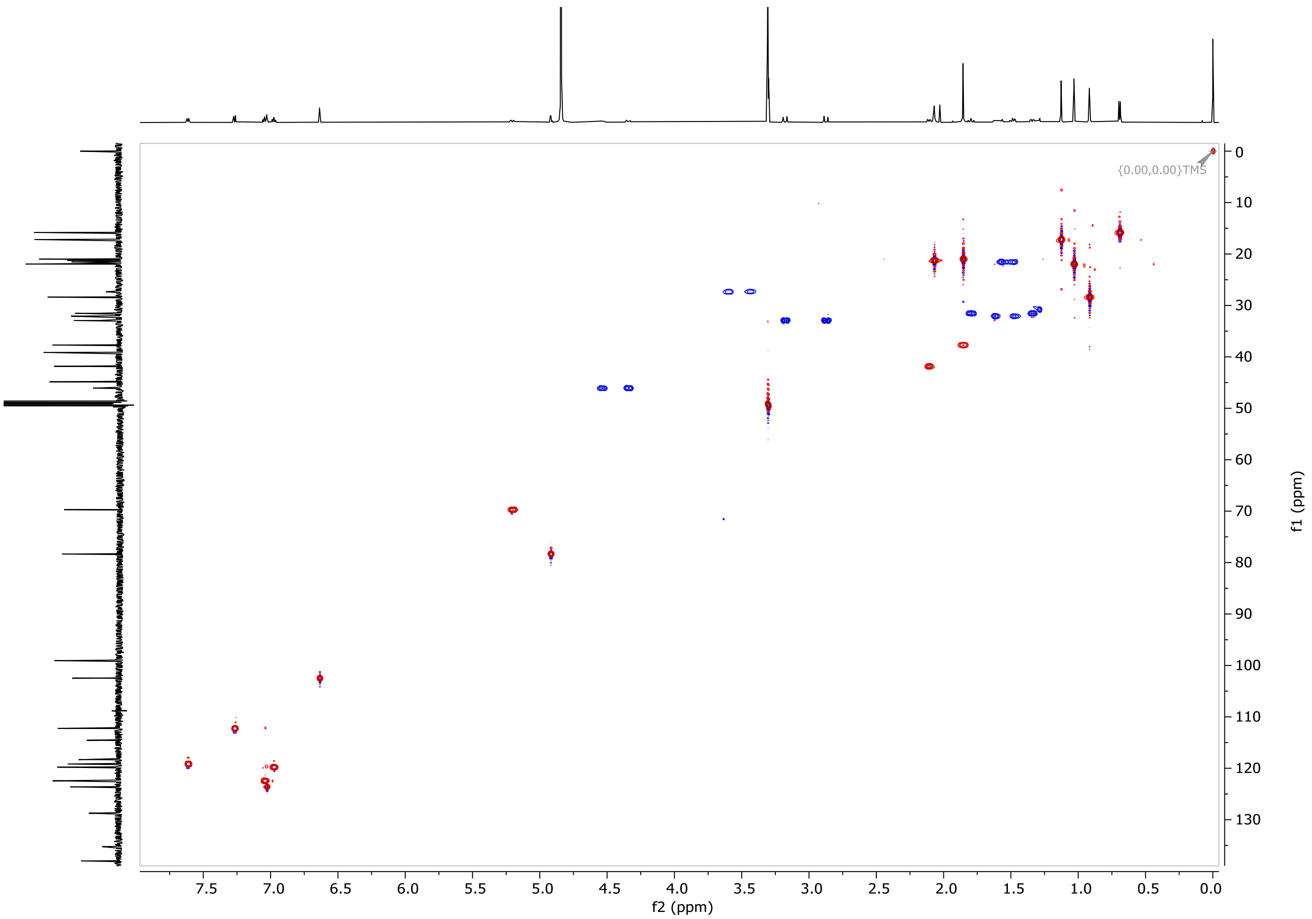

Figure S8. HSQC spectrum of ***2a*** in CD_3OD at 150 MHz and 600 MHz, red signals show CH and CH_3 groups, blue signals show CH_2 groups.

3 Acetoxy stachybotrylactam acetate-tryptophan 1 (*2b*)

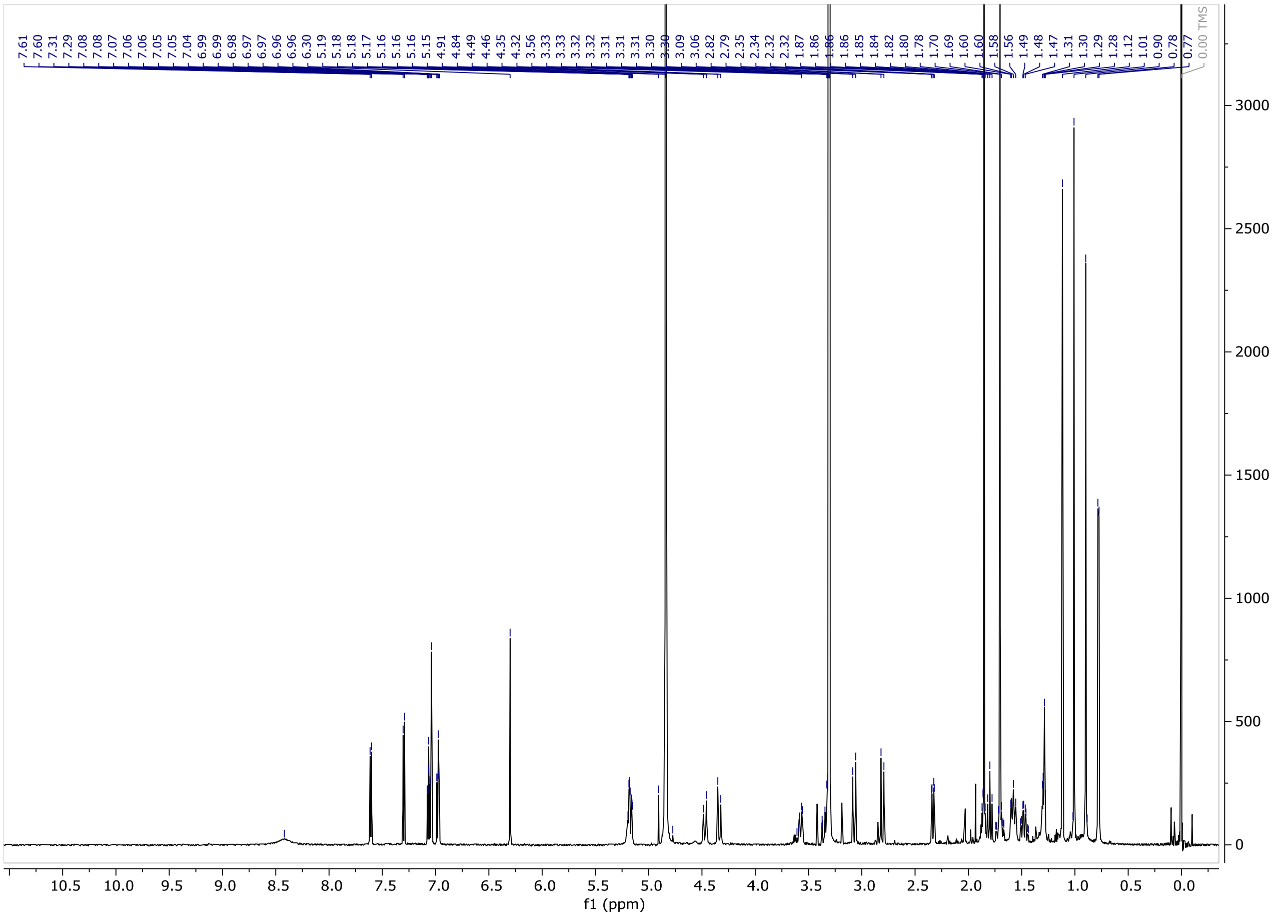

Figure S9. ^{1}H spectrum of ***2b*** in CD_3OD at 600 MHz.

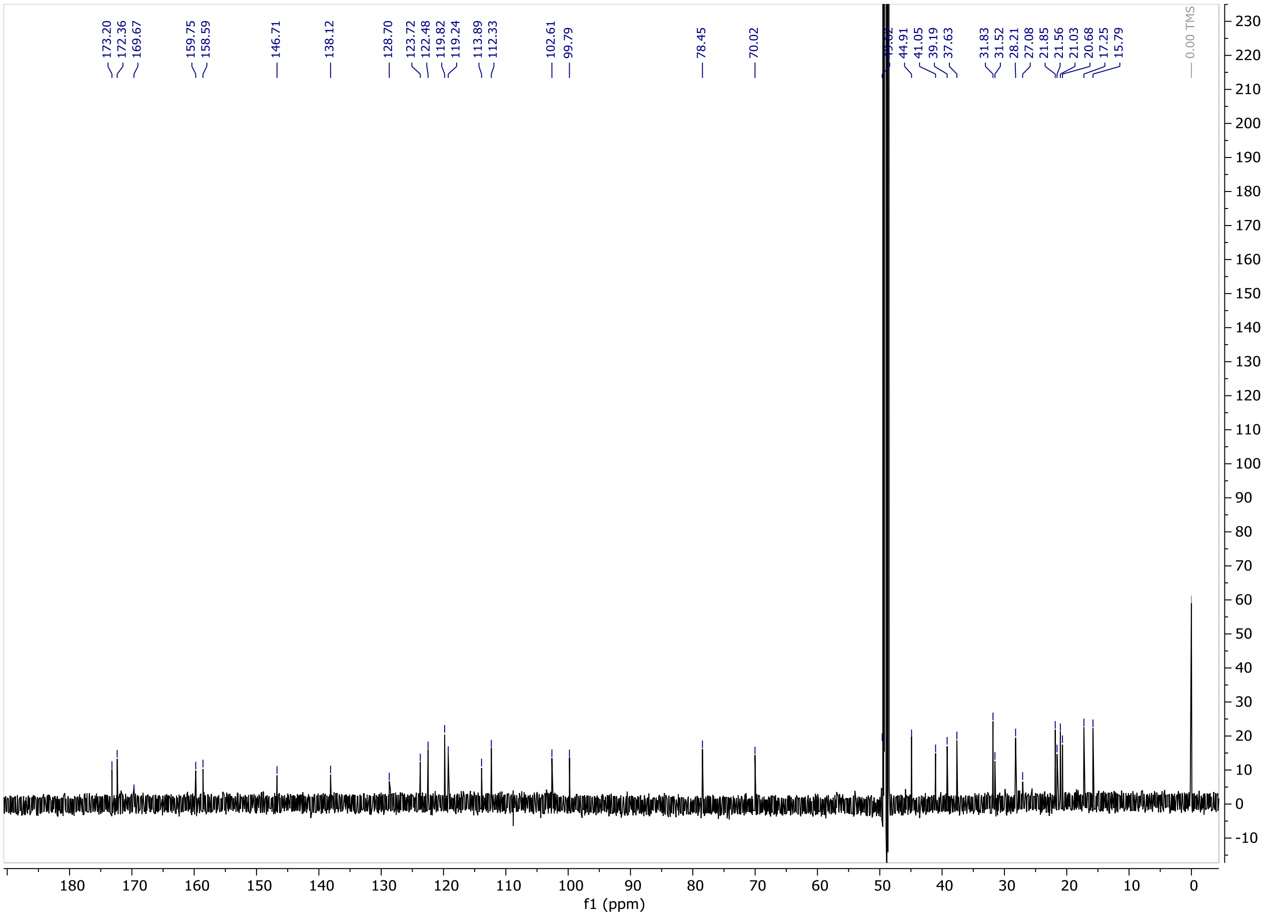

Figure S10. ^{13}C spectrum of ***2b*** in CD_3OD at 150 MHz.

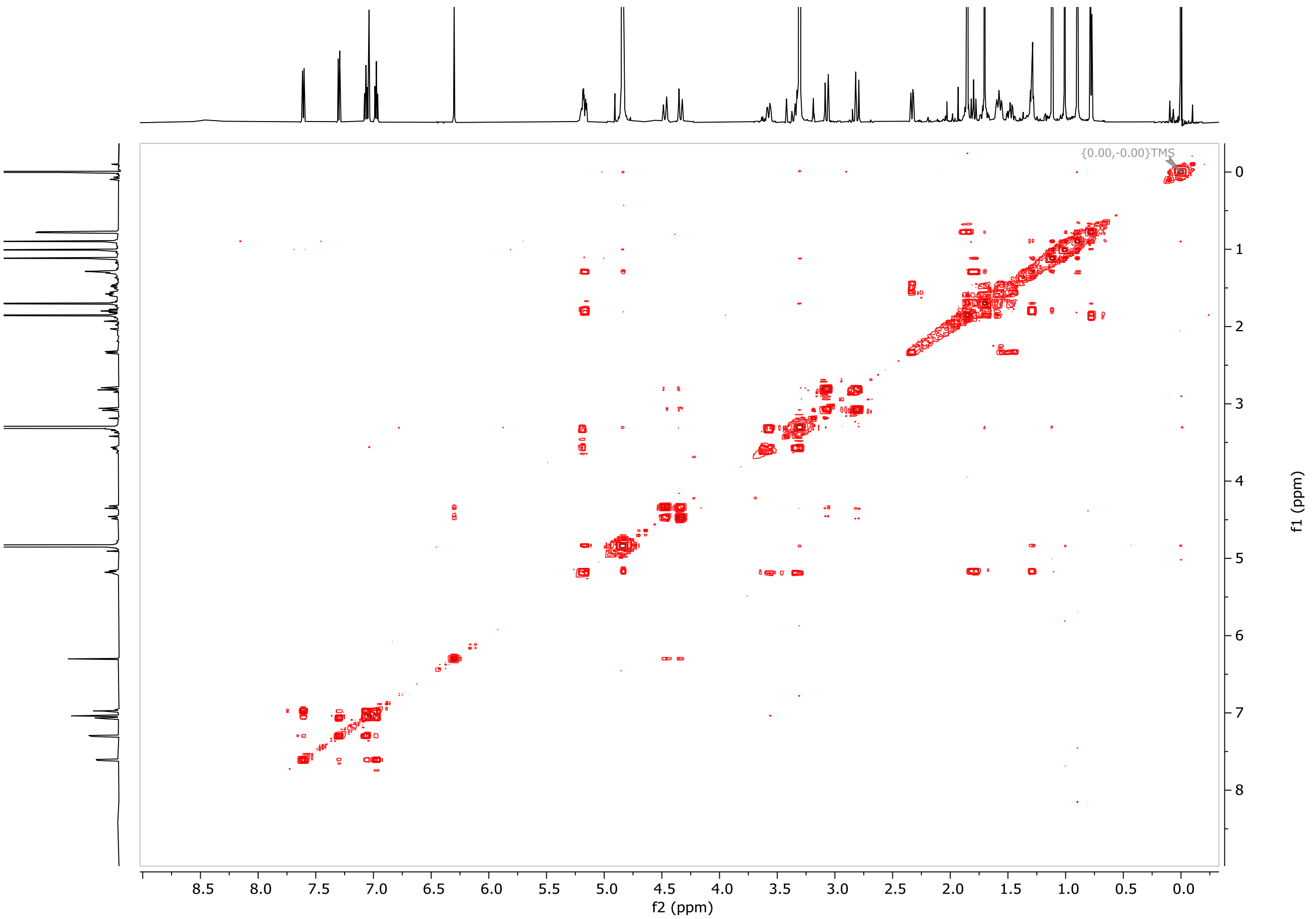

Figure S11. COSY spectrum of ***2b*** in CD_3OD at 600 MHz.

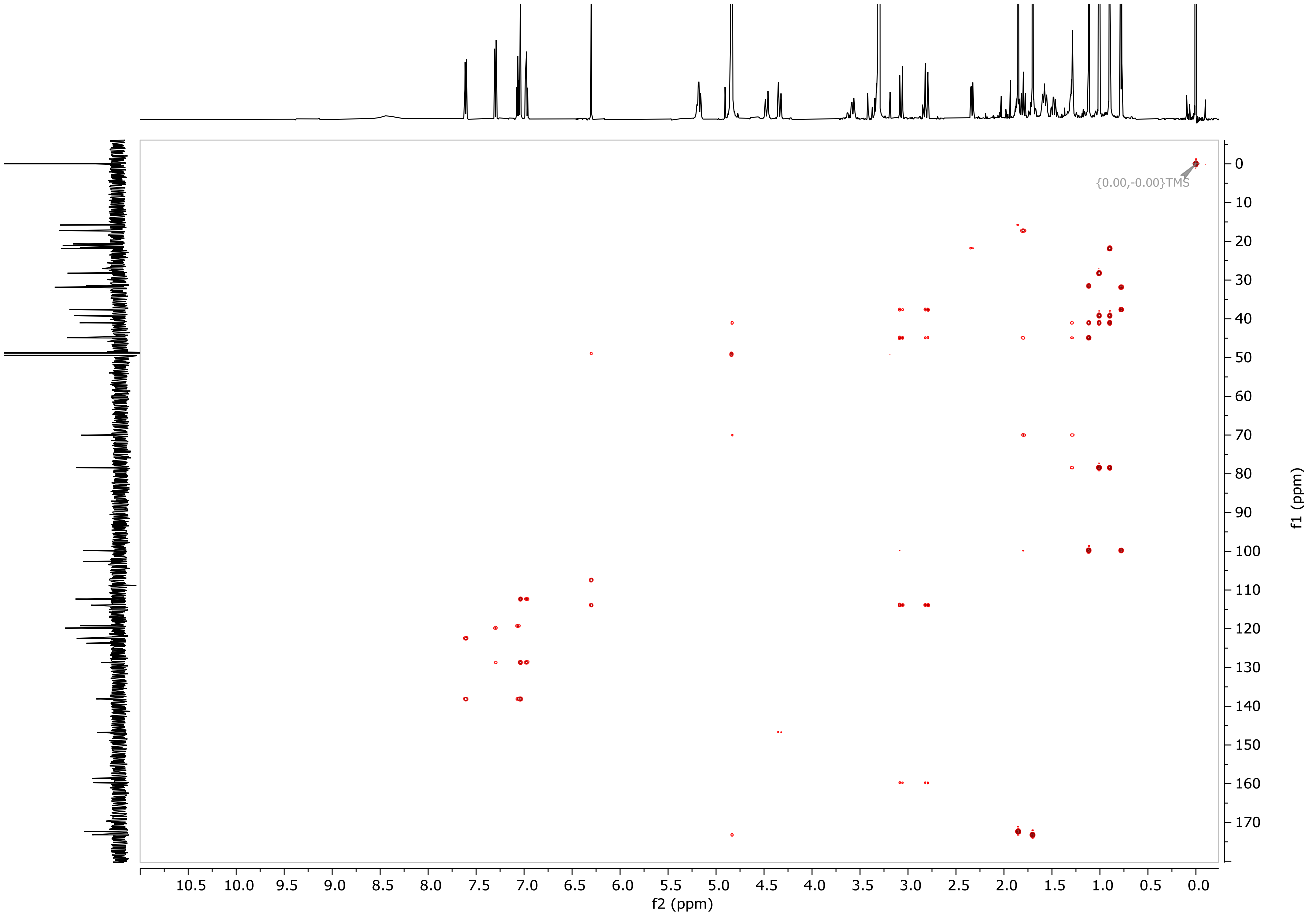

Figure S12. HMBC spectrum of ***2b*** in CD_3OD at 150 MHz and 600 MHz.

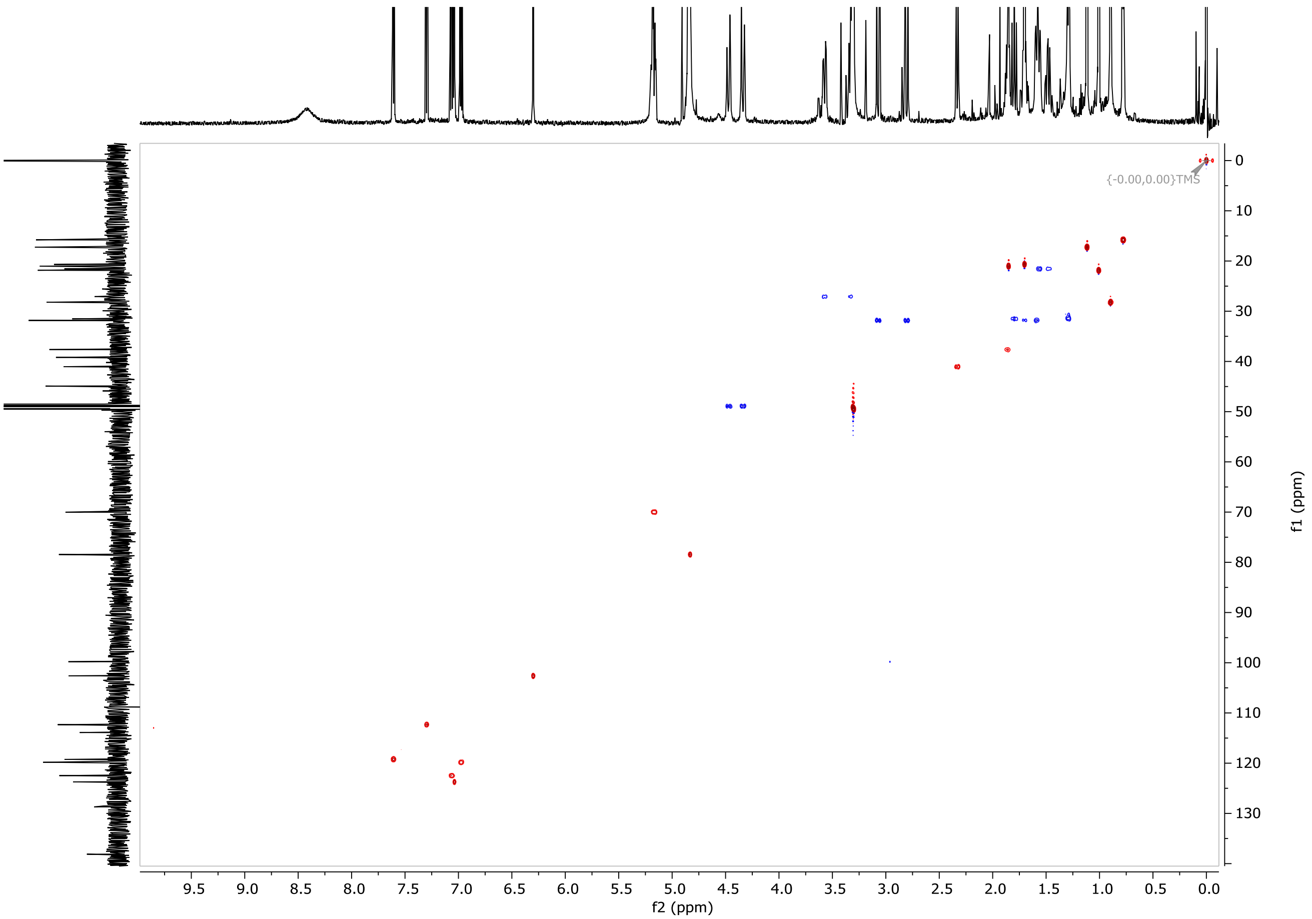

Figure S13. HSQC spectrum of ***2b*** in CD_3OD at 150 MHz and 600 MHz, red signals are CH and CH_3 groups, blue signals are CH_2 groups.

4 Acetoxy stachybotrylactam acetate-tryptamine 2 (*3a*)

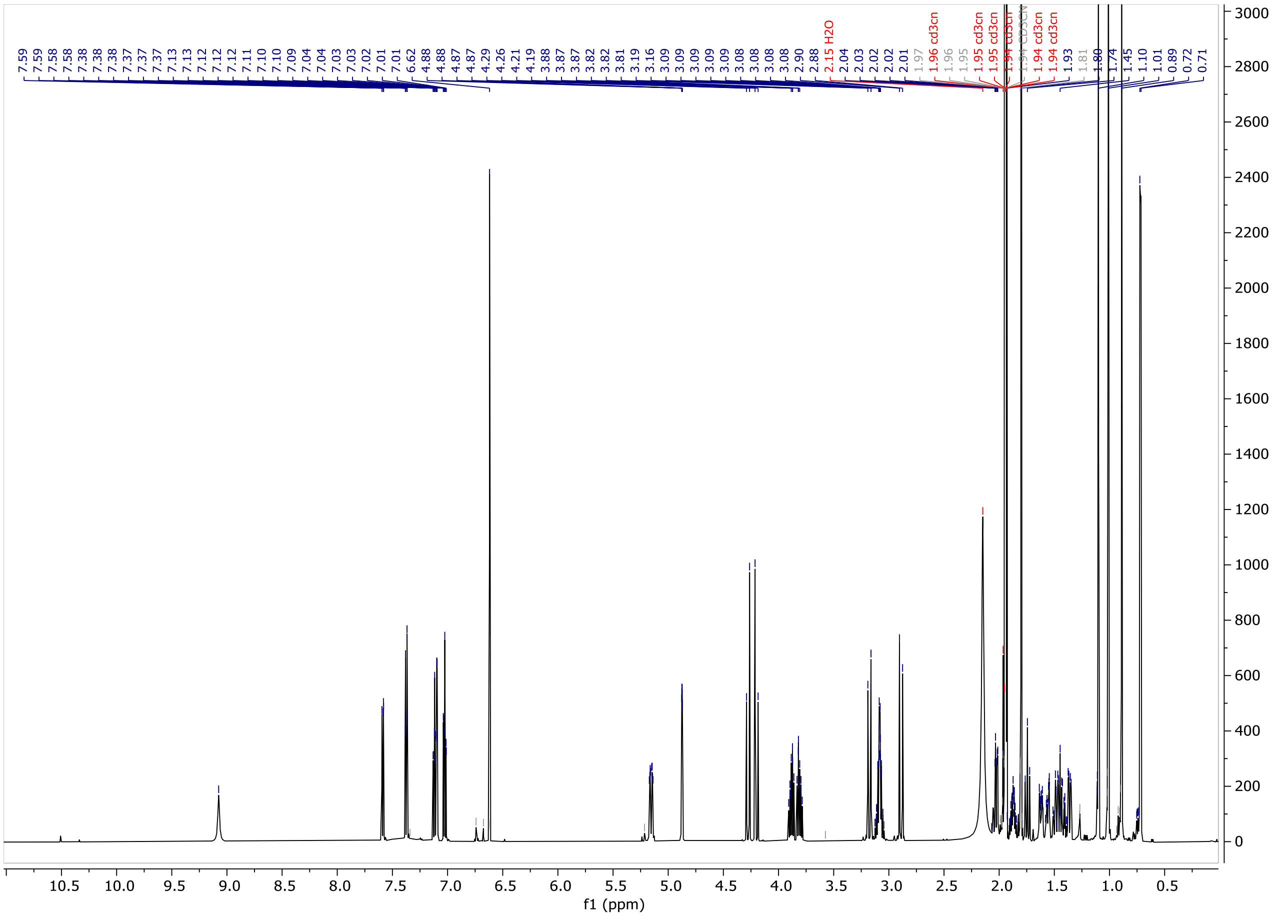

Figure S14. ^{1}H spectrum of ***3a*** in CD_3CN at 600 MHz.

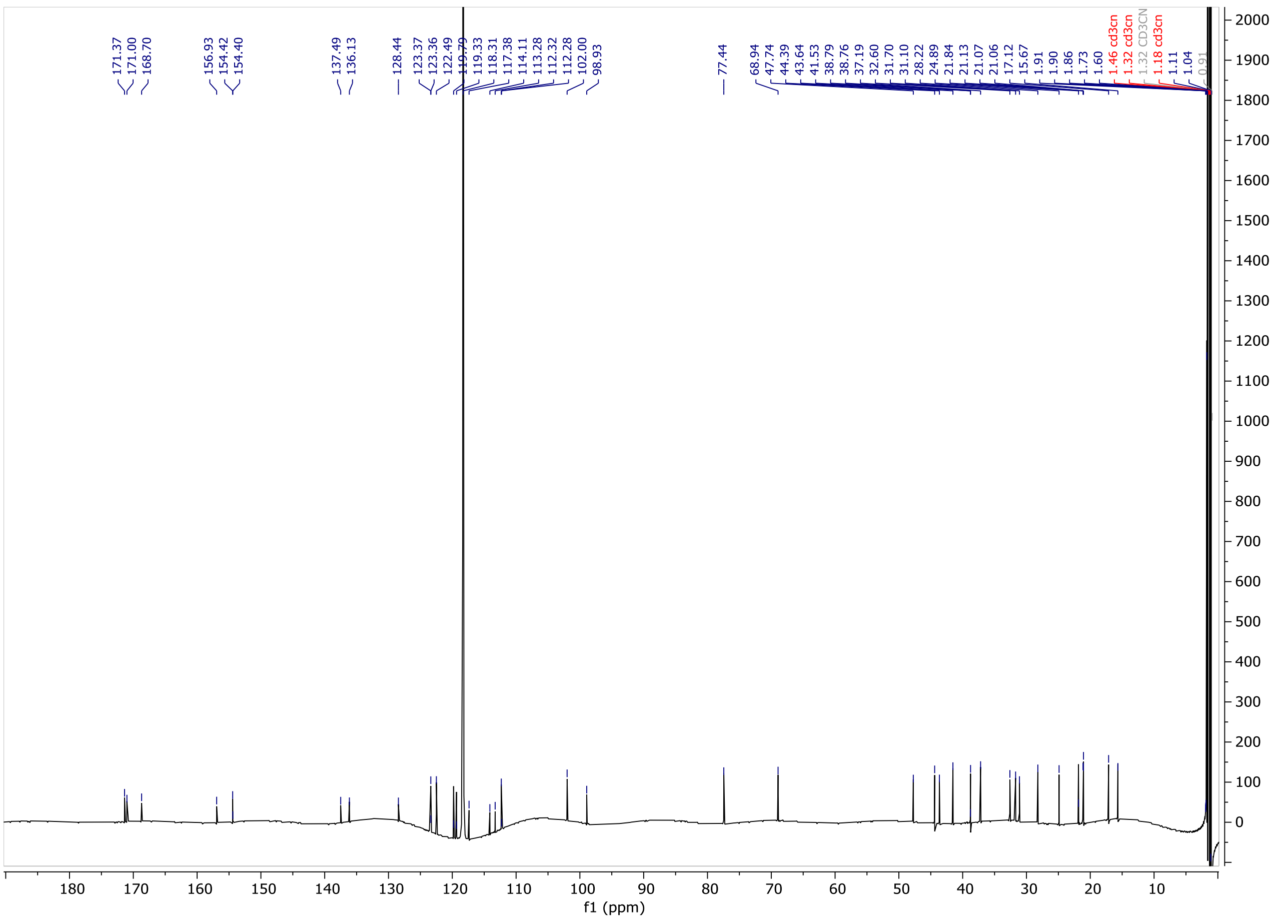

Figure S15. ^{13}C spectrum of ***3a*** in CD_3CN at 150 MHz.

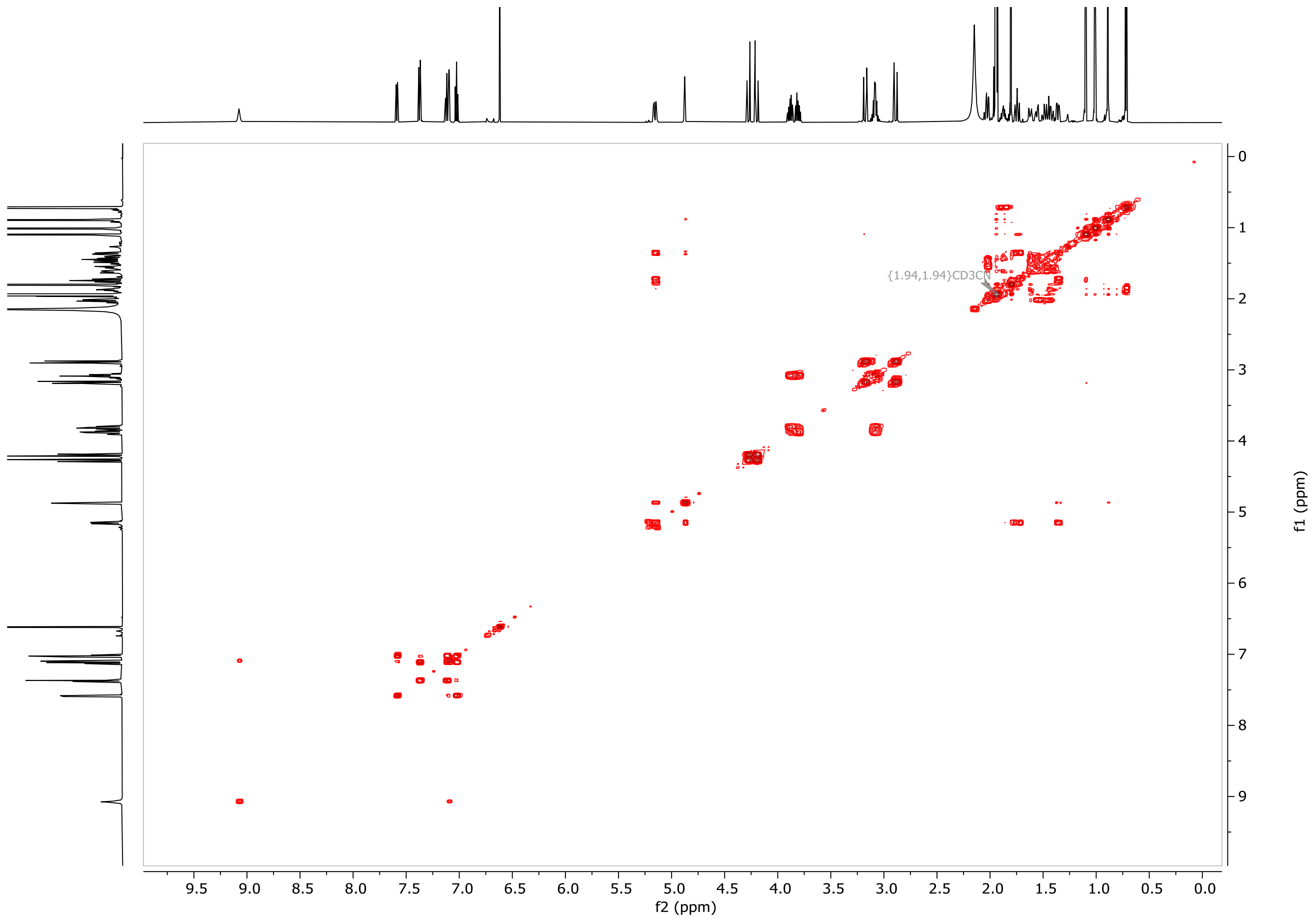

Figure S16. COSY spectrum of ***3a*** in CD_3CN at 600 MHz.

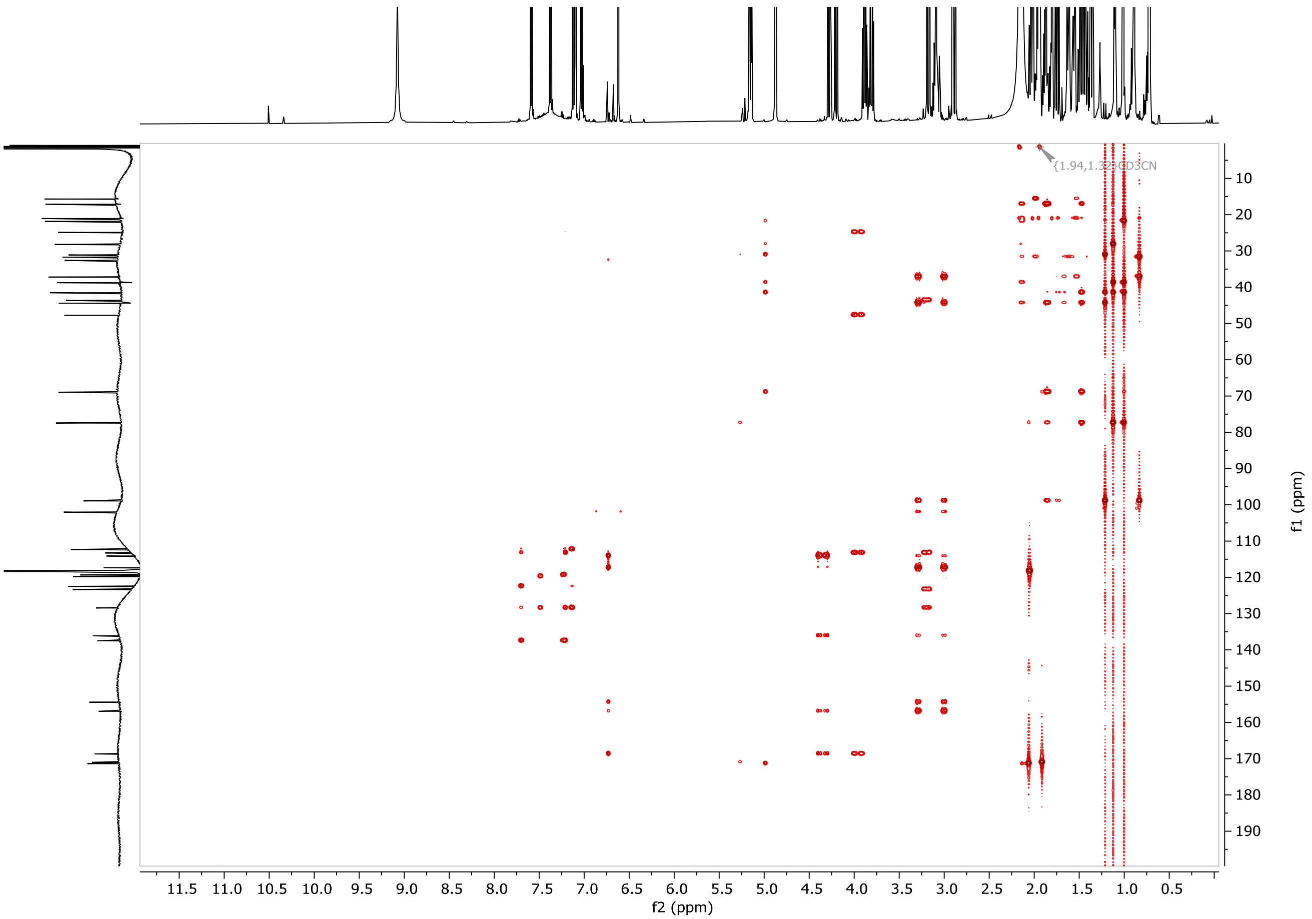

Figure S17. HMBC spectrum of ***3a*** in CD_3CN at 150 MHz and 600 MHz.

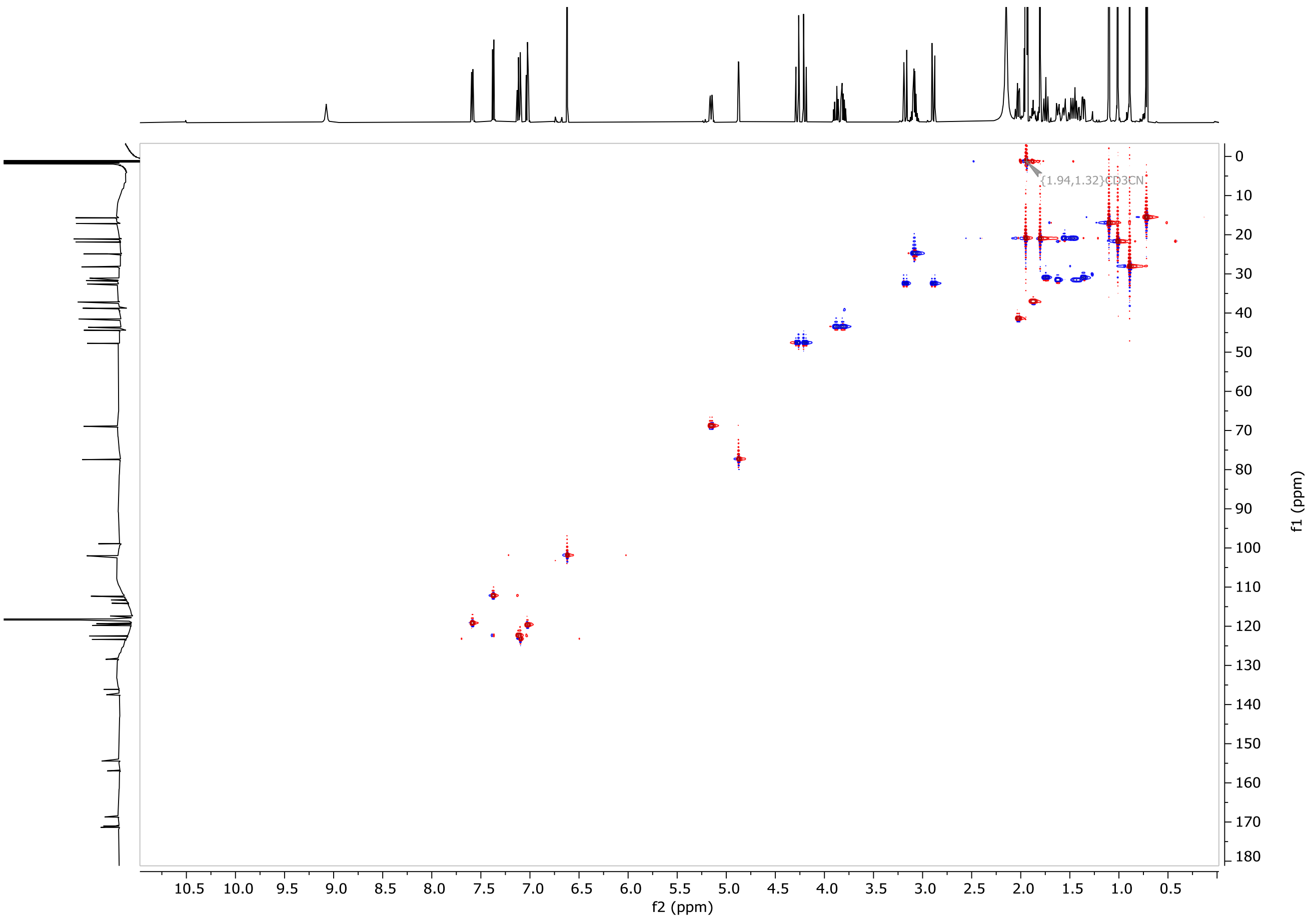

Figure S18. HSQC spectrum of ***3a*** in CD_3CN at 150 MHz and 600 MHz, red signals show CH and CH_3 groups, blue signals show CH_2 groups.

5 Acetoxy stachybotrylactam acetate-tryptamine 1 (*3b*)

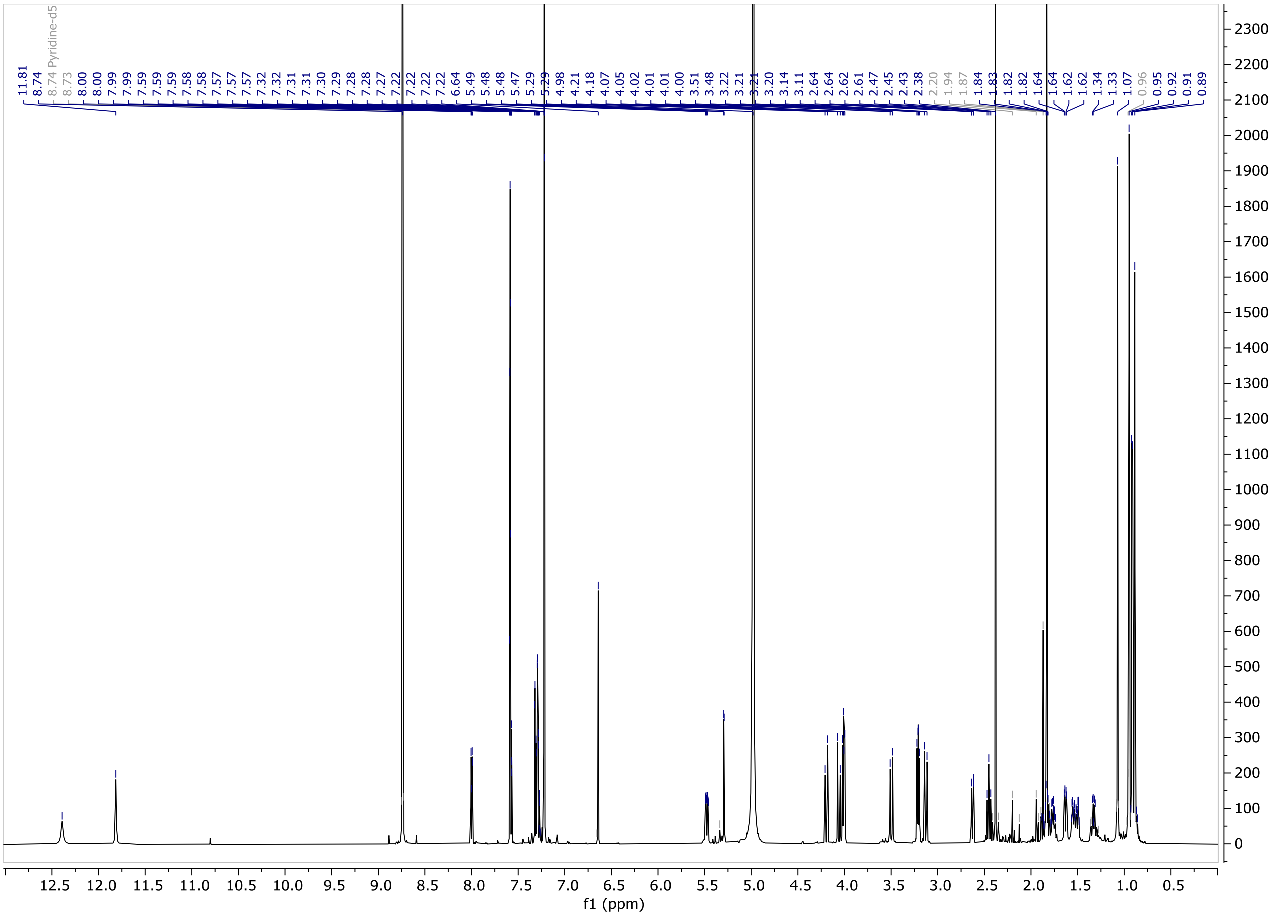

Figure S19. ^{1}H spectrum of ***3b*** in C_5D_5N at 600 MHz.

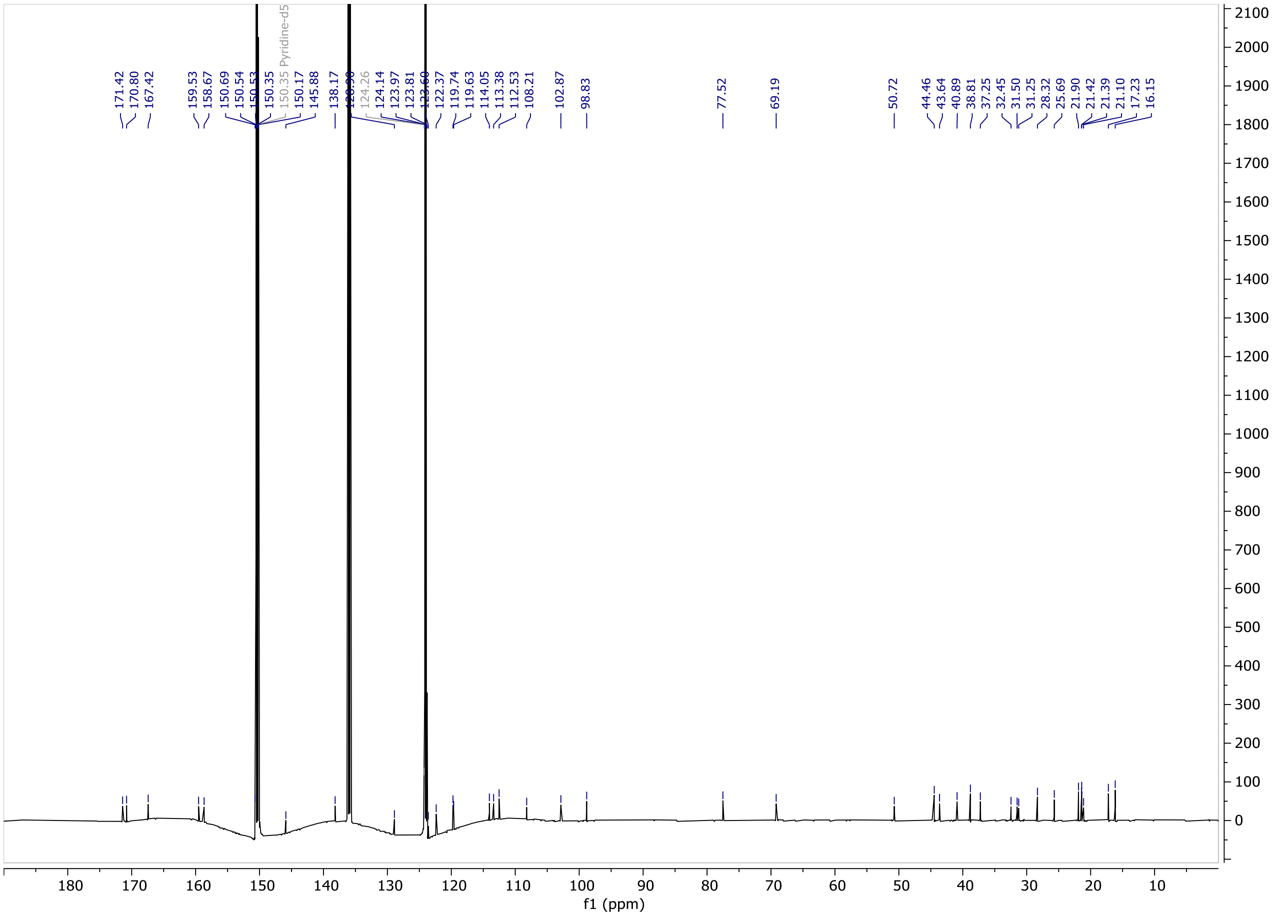

Figure S20. ^{13}C spectrum of ***3b*** in C_5D_5N at 150 MHz.

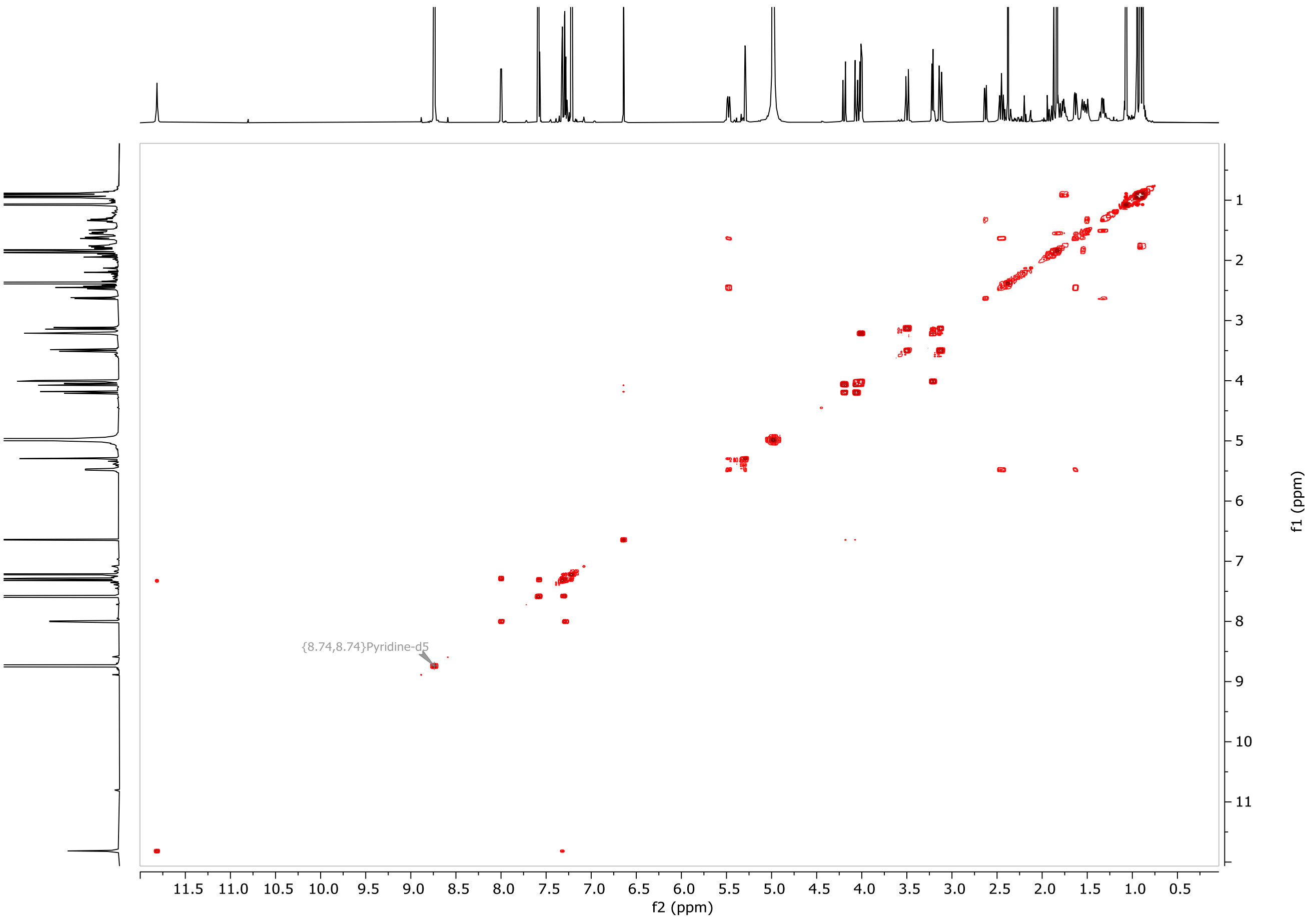

Figure S21. COSY spectrum of ***3b*** in C_5D_5N at 600 MHz.

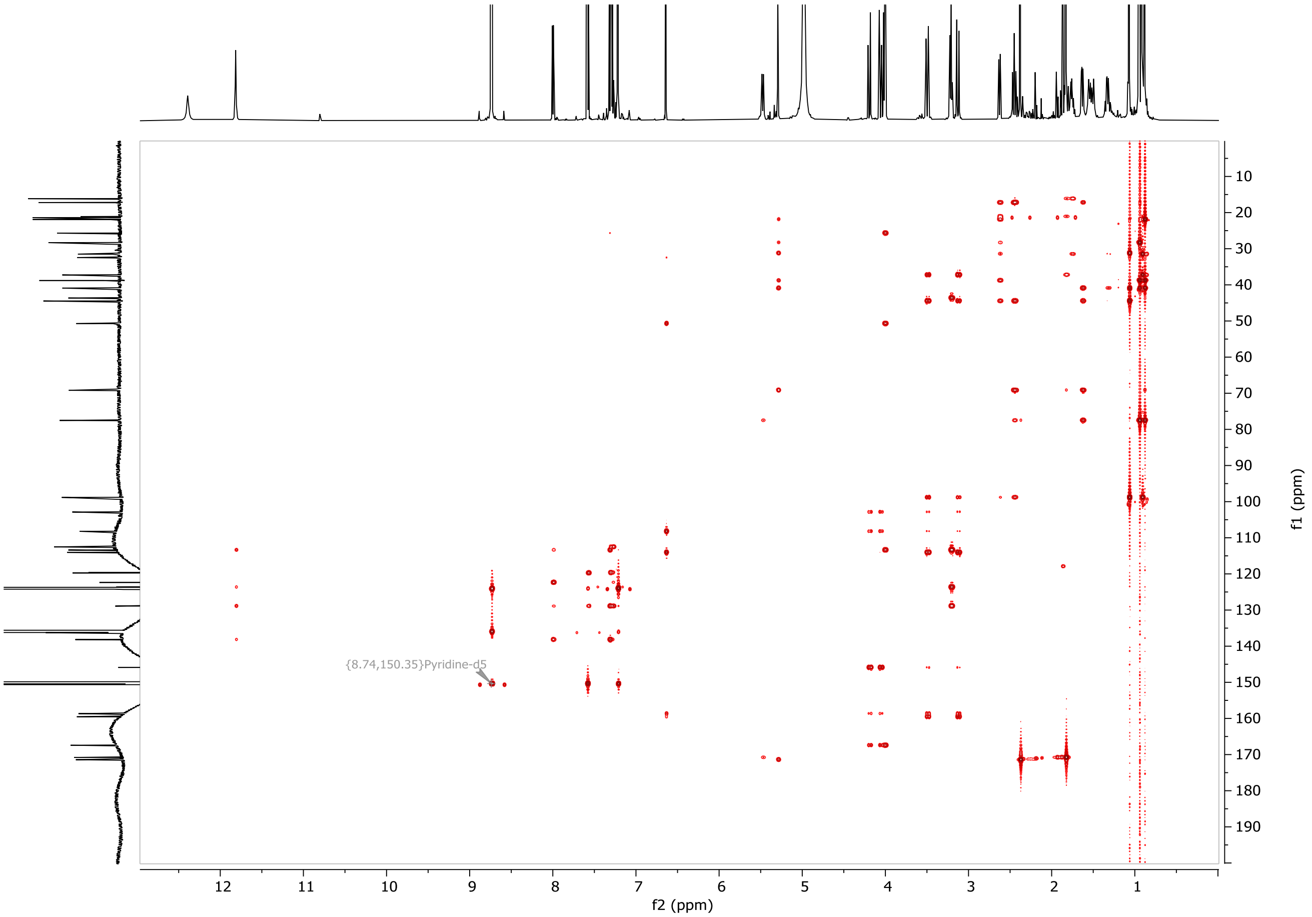

Figure S22. HMBC spectrum of ***3b*** in C_5D_5N at 150 MHz and 600 MHz.

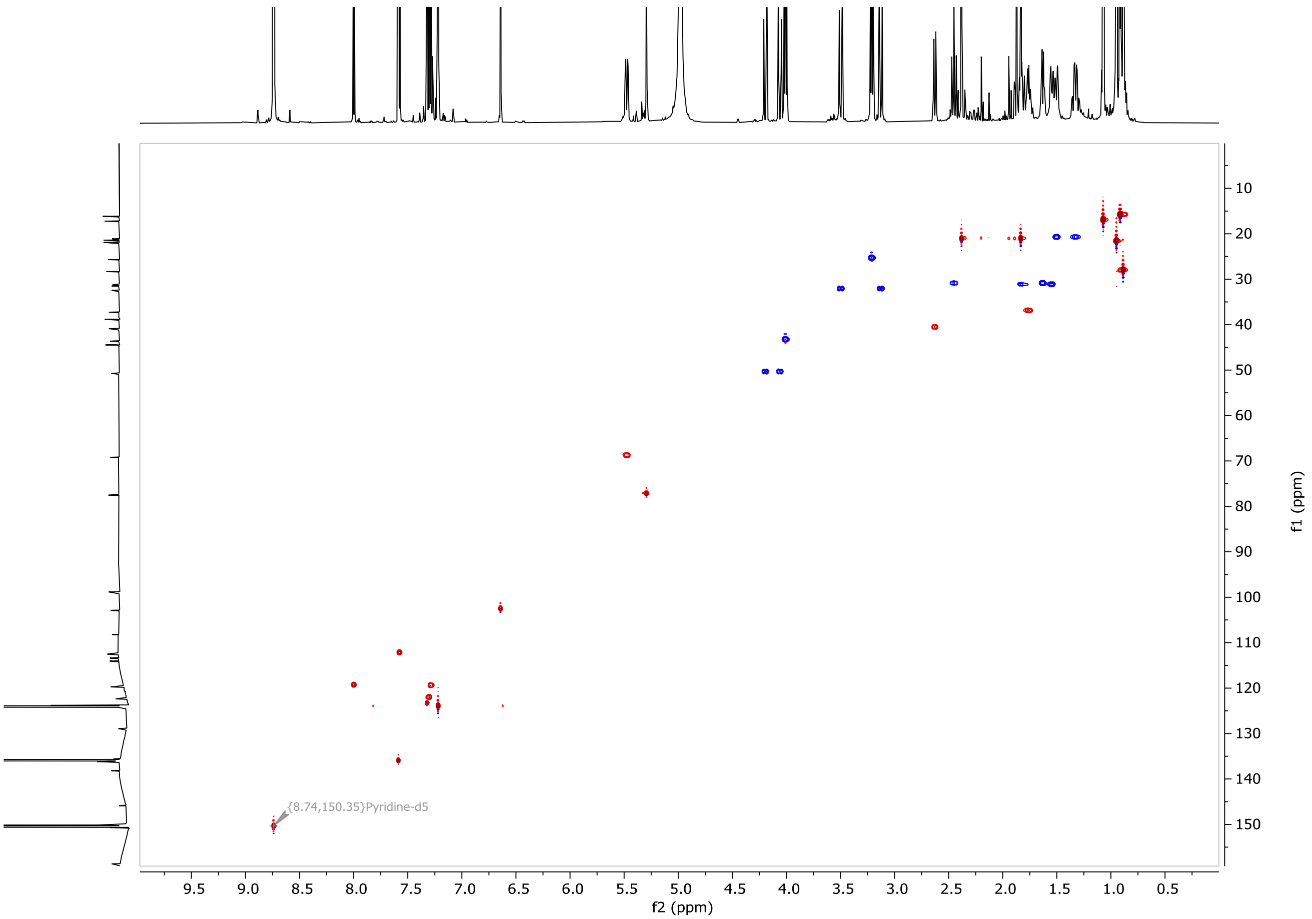

Figure S23. HSQC spectrum of ***3b*** in C_5D_5N at 150 MHz and 600 MHz, red signals show CH and CH_3 groups, blue signals show CH_2 groups.

6 Hepatic metabolism

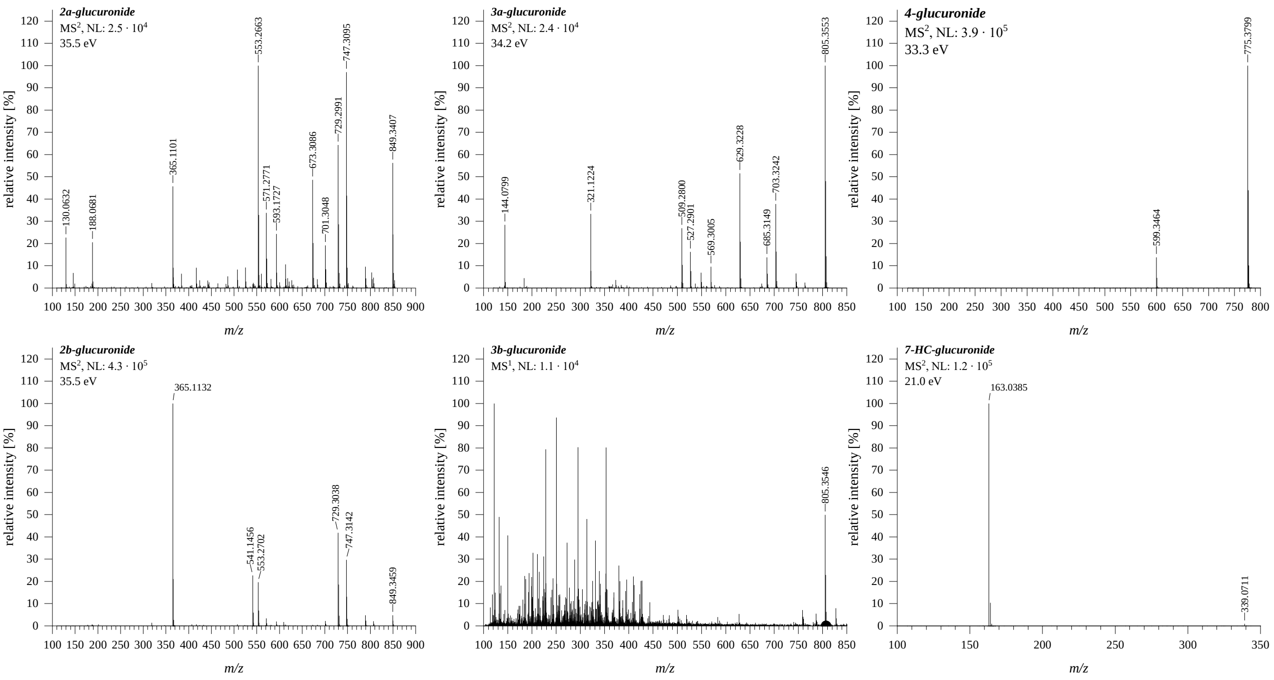

Figure S24. MS-spectra of glucuronized metabolites of the five tested compounds (***2a***, ***2b***, ***3a***, ***3b***, ***4***) and the positive control (7-hydroxycoumarin, ***7-HC***) obtained from human liver metabolism. Detection occurred with a HR-ESI-qTOF-MS in positive ionization mode at 4.5 kV capillary voltage and 0.5 kV end plate offset. Nebulizer gas was 2.0 bar, dry gas flow was 10 L/min. If applicable, fragmentation occurred in Auto-MS/MS mode selecting 3 precursors and exclusion after 3 spectra. Absolut threshold (1,000 sum) was 48 cts, applied collision energy is given for each compound specifically. Obtained MS^2 spectra were normalized to the most intense *m/z* (NL: normalization level) to give the relative intensity in %. A neutral loss of 176.0321 indicated the cleavage of the glucuronide.

Table S2. Overview of *m/z* assigned to the glucuronized metabolite of the five tested compounds (***2a***, ***2b***, ***3a***, ***3b***, ***4***) and the positive control (***7-HC***) obtained from human liver metabolism. Detection occurred with a HR-ESI-qToF-MS in positive ionization mode. Shown are the *m/z* values of the parent ion's proton adduct [M+H] with the mass error (Δm) in ppm, fragmentation level (MS^n), collision energy (CE) applied in eV, the detected fragment *m/z* and the Δm of each fragment ion, n.d.= not detected, n.a.= not applicable.

Metabolite	Parent ion *m/z* $[M+H]^+$ ± Δm	MS^n	CE [eV]	Glucuronide fragment *m/z* $[M+H]^+$	Δm [ppm]
2a-glucuronide	849.3407 ± 4.6	2	35.5	673.3086	5.9
2b-glucuronide	849.3459 ± 1.5	2	35.5	n.d.	n.a.
3a-glucuronide	805.3553 ± 0.6	2	34.2	629.3228	-0.3
3b-glucuronide	805.3546 ± 0.3	1	n.a.	n.a.	n.a.
4-glucuronide	775.3799 ± 4.3	2	33.3	599.3464	-3.2
7-HC-glucuronide	339.0711 ± 3.1	2	21.0	163.0390	3.2

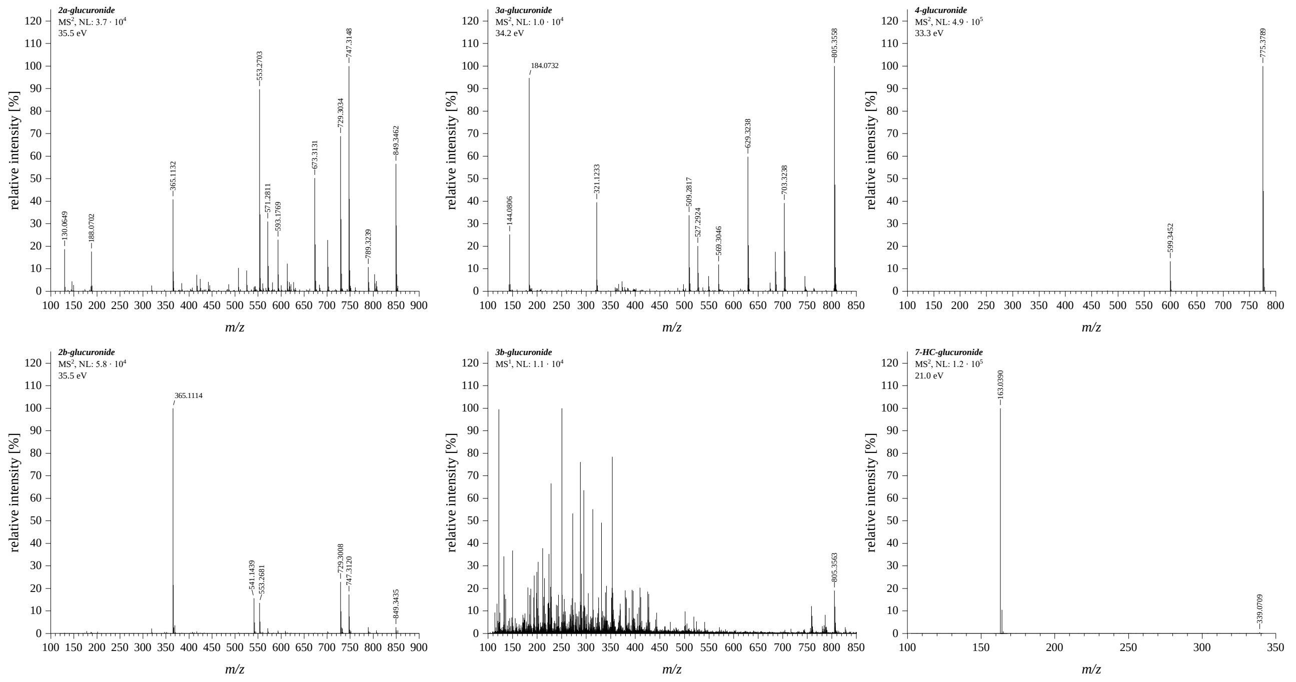

Figure S25. MS-spectra of glucuronized metabolites of the five tested compounds (***2a***, ***2b***, ***3a***, ***3b***, ***4***) and the positive control (7-hydroxycoumarin, ***7-HC***) obtained from horse liver metabolism. Detection occurred with a HR-ESI-qTOF-MS in positive ionization mode at 4.5 kV capillary voltage and 0.5 kV end plate offset. Nebulizer gas was 2.0 bar, dry gas flow was 10 L/min. If applicable, fragmentation occurred in Auto-MS/MS mode selecting 3 precursors and exclusion after 3 spectra. Absolut threshold (1,000 sum) was 48 cts, applied collision energy is given for each compound specifically. Obtained MS^2 spectra were normalized to the most intense *m/z* (NL: normalization level) to give the relative intensity in %. A neutral loss of 176.0321 indicated the cleavage of the glucuronide.

Table S3. Overview of *m/z* assigned to the glucuronized metabolite of the five tested compounds (***2a***, ***2b***, ***3a***, ***3b***, ***4***) and the positive control (***7-HC***) obtained from horse liver metabolism. Detection occurred with a HR-ESI-qToF-MS in positive ionization mode. Shown are the *m/z* values of the parent ion's proton adduct [M+H] with the mass error (Δm) in ppm, fragmentation level (MS^n), collision energy (CE) applied in eV, the detected fragment *m/z* and the Δm of each fragment ion, n.d.= not detected, n.a.= not applicable.

Metabolite	Parent ion *m/z* $[M+H]^+$ ± Δm	MS^n	CE [eV]	Glucuronide fragment *m/z* $[M+H]^+$	Δm [ppm]
2a-glucuronide	849.3462 ± 1.9	2	35.5	673.3131	-0.7
2b-glucuronide	849.3459 ± 1.5	2	35.5	n.d.	n.a.
3a-glucuronide	805.3558 ± 1.2	2	34.2	629.3238	-1.9
3b-glucuronide	805.3563 ± 1.8	1	n.a.	n.a.	n.a.
4-glucuronide	775.3789 ± 3.0	2	33.3	599.3452	-1.2
7-HC-glucuronide	339.0709 ± 3.7	2	21.0	629.3238	3.2
